# An Overview of Systematic Reviews of Herbal Medicine for Irritable Bowel Syndrome

**DOI:** 10.3389/fphar.2022.894122

**Published:** 2022-05-18

**Authors:** Hyejin Jun, Seok-Jae Ko, Keumji Kim, Jinsung Kim, Jae-Woo Park

**Affiliations:** ^1^ Department of Internal Korean Medicine, Kyung Hee University Hospital at Gangdong, Seoul, South Korea; ^2^ Department of Clinical Korean Medicine, Graduate School of Kyung Hee University, Seoul, South Korea; ^3^ Department of Gastroenterology, College of Korean Medicine, Kyung Hee University, Seoul, South Korea

**Keywords:** herbal medicine, irritable bowel syndrome, overview, systematic reviews, metaanalysis

## Abstract

**Background:** Irritable bowel syndrome (IBS) is a common disorder with abdominal pain and bowel habits changes. Herbal medicines (HMs) are frequently used in the treatment of IBS. Therefore, several systematic reviews (SRs) have been conducted to assess the efficacy and safety of HM in IBS patients. This study aimed to investigate the methodology and quality of evidence of SRs, and to describe the current state of research and evidence for the treatment of IBS with HM.

**Methods:** SRs published up to January 2022 were searched using six electronic databases. SRs and/or meta-analyses on the use of HMs for IBS were included. The effects of placebo, conventional medicine (CM), and probiotics were compared with those of HMs. Two investigators independently extracted the data and assessed methodological quality using the Measure Tool to Assessment System Reviews 2 (AMSTAR 2). Grading of Recommendations, Assessment, Development, and Evaluation (GRADE) was used to evaluate the quality of evidence for each main finding.

**Results:** Eighteen SRs were included in this overview. Among them, eight SRs reported only specific subtypes of IBS: six SRs reported patients with diarrhea-predominant IBS, and two SRs reported patients with constipation-predominant IBS. In terms of total efficacy, HM was more effective than placebo, CM, or probiotics. HM showed a more significant effect than CM in relieving independent IBS symptom score (abdominal pain score, diarrhea score, abdominal distension score, stool frequency score, etc.) and recurrence rate. The rate of adverse events was significantly lower with HM compared to CM, and no serious adverse events were reported with HM treatment. According to AMSTAR 2, the methodological quality of the included SRs was extremely low. Furthermore, the quality of evidence for total efficacy was considered low or very low according to the GRADE tool.

**Conclusion:** HM can be considered as an effective and safe treatment for IBS. However, the methodological quality of the included SRs and the quality of evidence was generally low. Therefore, well-designed randomized controlled trials are needed in the future so that a high-quality SR can be used to better assess the safety and efficacy of HM in the treatment of IBS.

**Systematic Review Registration:** https://osf.io/nt6wz, identifier 10.17605/OSF.IO/NT6WZ.

## 1 Introduction

Irritable bowel syndrome (IBS) is a functional bowel disorder characterized by recurrent abdominal pain associated with changes in bowel habits (Lacy et al., 2016). The worldwide prevalence of IBS is 11.2% (Lovell and Ford, 2012). According to the Rome IV criteria, IBS is diagnosed when a patient experiences, on average, at least 1 day per week in the last 3 months, recurrent abdominal pain that is, associated with symptoms such as defecation, change in frequency of stool, and form of stool (Hellström and Benno, 2019). Additionally, IBS is categorized into four subtypes based on the predominant bowel habits: IBS with predominant constipation (IBS-C), IBS with predominant diarrhea (IBS-D), IBS with mixed bowel habits (IBS-M), and unclassified IBS (IBS-U) (Lacy et al., 2016). The pathophysiology of IBS remains unclear, although it is known to be caused by the dysregulation of gut motility, visceral hypersensitivity, intestinal microbiomes, inflammation, food-related sensitivity, genetics, and psychosocial dysfunction (Defrees and Bailey, 2017). The primary treatment for IBS includes lifestyle modification and education, such as diet and exercise. Depending on the symptoms, medications such as antispasmodics, antidiarrheal drugs, laxatives, and 5-hydroxytryptamine 3 receptor antagonists may be used (Defrees and Bailey, 2017). However, such medications do not adequately improve symptoms and quality of life; also, side effects may occur. Consequently, there is growing demand for complementary and alternative medicine (CAM) treatments for patients with IBS (Hawrelak et al., 2020). Herbal medicine (HM) is the most common CAM modality used in IBS patients (Bahrami et al., 2016). HM is based on the use of medicinal plants for the prevention and treatment of diseases (Firenzuoli and Gori, 2007) and has been used in Asian countries, including Korea, China, Iran, and Japan, for a long time. Several studies have reported the efficacy and safety of HM for IBS; however, the diverse results from previous systematic reviews (SRs) make it difficult to make firm conclusions regarding the application of HM for IBS. Therefore, we aimed to conduct an overview of SRs on the efficacy and safety of HM in IBS. Also, we aimed to assess the methodological quality and quality of evidence of SRs, and consider how research in this field should proceed in the future.

## 2 Methods

The protocol for this overview has been published previously (Jun et al., 2021). Ethical approval was not required because this is an overview of SRs.

### 2.1 Criteria for Selecting Reviews for Inclusion

#### 2.1.1 Types of Studies

SRs that estimated the efficacy and safety of HM for the treatment of IBS were included. SRs consisting of randomized controlled trials (RCTs) with a meta-analysis or not were included. SRs including animal studies were excluded.

#### 2.1.2 Types of Participants

Studies that included patients with IBS, regardless of age, sex, or race, and diagnosed using the Rome or other criteria, were included.

#### 2.1.3 Types of Interventions

Studies involving any type of oral HM, either an original composition or a modified one with some herbs added or removed, that were used as an intervention, regardless of dosage, were included. The preparation of HM was not restricted; decoctions and granules were mostly used in the included SRs. SRs that only involved a single herbal extract for the intervention were excluded because herbal prescriptions are mainly used in the clinical field. SRs that involved both of a single herbal extract and herbal prescriptions were included. A placebo of HM, conventional treatment such as Western medication, and probiotics were used as the controls.

#### 2.1.4 Types of Outcome Measures

The primary outcome measure was the total efficacy rate (TER). The secondary outcomes included the individual symptom score of IBS (abdominal pain score, diarrhea score, abdominal distention score, frequency of defecation score, and fecal property score), IBS symptom severity score (IBS-SSS), total symptom score, stool form, recurrence rate after treatment, adverse event rate, pain threshold, defecation threshold, and IBS quality of life (IBS-QoL).

### 2.2 Search Strategy

Two reviewers (HJ and KK) conducted a comprehensive search of four English databases (Medline via PubMed, Excerpta Medica database, Cochrane Database of Systematic Reviews, and Allied and Complementary Medicine Database), one Korean database (Oriental Medicine Advanced Searching Integrated System), and one Chinese database (China National Knowledge Infrastructure database) from their inception dates to January 2022. The search strategy for Medline is shown in Table 1. Modified search strategies were applied to the other databases. The search date was January 11, 2022, and there were no language restrictions. If only a part of the SR met the inclusion criteria of this overview, we extracted only that part.

**TABLE 1:** Search strategy used in Medline.

#1. Irritable bowel syndrome [mh]
#2. traditional Chinese medicine [tiab]
#3. herbal medicine [tiab]
#4. herb*[tiab]
#5. systematic review [tiab]
#6. meta-analysis [tiab]
#7. #2 OR #3 OR #4
#8. #5 OR #6
#9. #1 AND #7 AND #8

### 2.3 Study Selection and Data Extraction

#### 2.3.1 Selection of Studies

**FIGURE 1:**
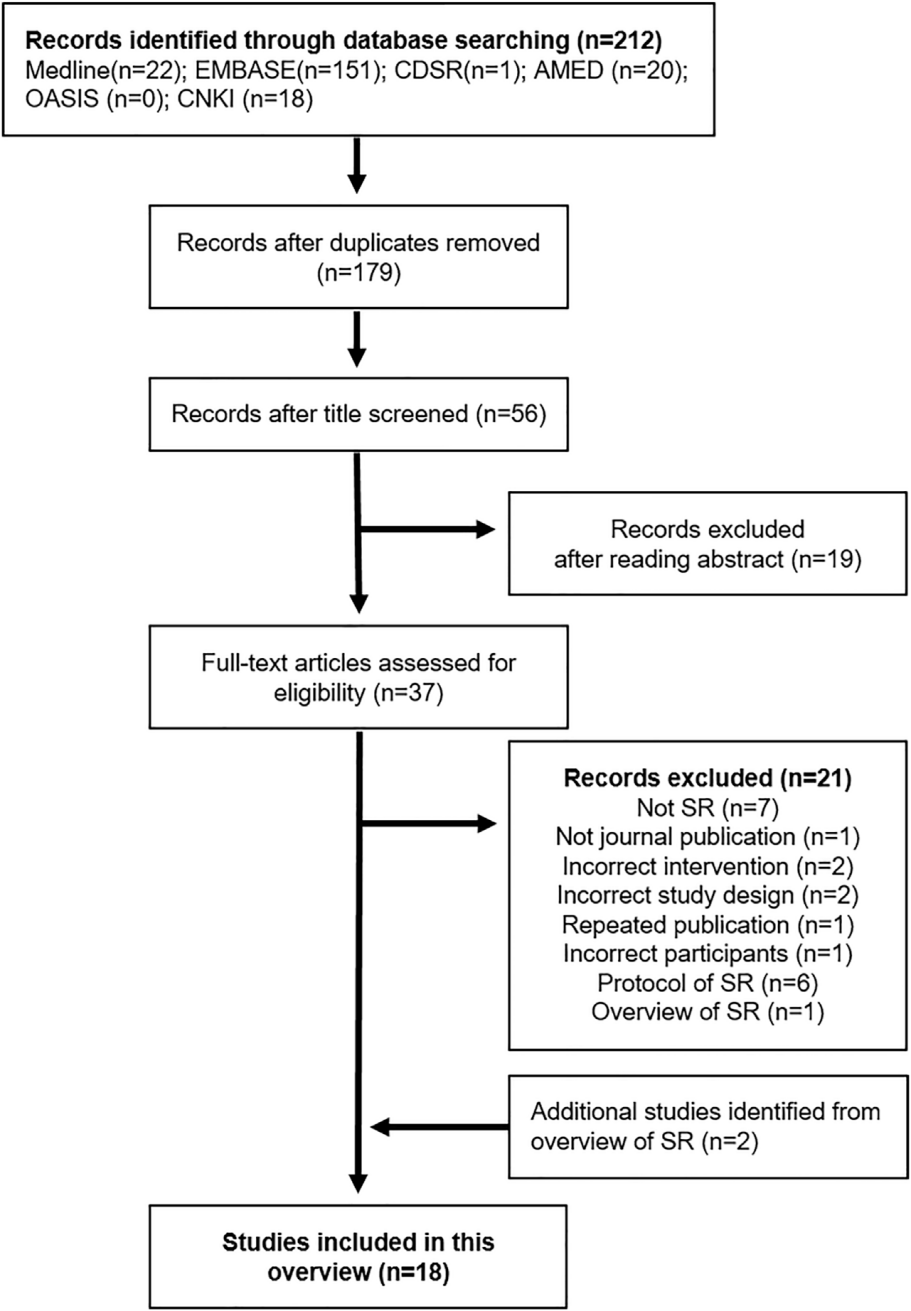
Flow chart of the study selection process. EMBASE: Excerpta Medica database, CDSR: Cochrane Database of Systematic Reviews, AMED: Allied and Complementary Medicine Database, OASIS: Oriental Medicine Advanced Searching Integrated System, CNKI: China National Knowledge Infrastructure database, SR: Systematic review.

Two reviewers independently reviewed the titles and abstracts of studies that met the inclusion criteria. The reasons for exclusion and the number of excluded studies were reported using a PRISMA flow chart (Figure 1). If necessary, the mediator (JWP) intervened and resolved any disagreements.

#### 2.3.2 Data Extraction and Management

Two reviewers independently extracted the data and wrote the standard data extraction form, which included basic study information, such as the first author, publication year, written language, number of included studies and patients, IBS subtype, interventions, control, main results (meta-analysis), and reported adverse events. Any disagreement was resolved through a discussion with the mediator. Microsoft Excel 2019 (Microsoft Corp, Redmond, WA, United States) was used to extract data.

### 2.4 Quality Assessment

Two reviewers assessed the methodological quality of the included SRs using a measurement tool to assess SRs 2 (AMSTAR 2) (Shea et al., 2017). This is a validated tool that comprises 16 items, the responses of which can be “yes,” “partially yes,” or “no.” AMSTAR 2 is a domain-based rating system with seven critical domains and nine noncritical domains, as opposed to the original AMSTAR. Thus, rather than generating a total score, AMSTAR 2 assesses the overall confidence of each SR as “high,” “moderate,” “low,” or “critically low,” based on critical and non-critical domain performance which are weighted differently in the rating rules (Shea et al., 2017). The two reviewers discussed and resolved any arguments, and, if necessary, the mediator intervened.

### 2.5 Data Analysis

For qualitative synthesis, meta-analysis data from each SR were extracted in the form of odds ratio (OR) or risk ratio (RR) for dichotomous data, and in the form of mean difference (MD) or standardized mean difference (SMD) for continuous data with 95% confidence intervals (CIs). The heterogeneity of each included SR was extracted, which was detected by I-squared (*I* ^2^) statistics and chi-squared (*χ* ^2^) tests. No re-analysis of the data was performed using a meta-analysis approach because of the insufficient number of trials sharing identical herbal prescriptions or comparators. Moreover, because the purpose of this overview was to summarize and assess the related SRs reported to date, the reviewers decided not to re-analyze them.

### 2.6 Quality of Evidence Assessment

According to the protocol, it was planned to extract the grading of recommendations, assessment, development, and evaluation (GRADE) assessment of the included SRs as Cochrane handbook recommended (Pollock et al., 2021). However, only two SRs (Bu et al., 2020; Wang et al., 2020) reported the GRADE assessment results, we analyzed the overall quality of evidence for the included SRs using the GRADE tool. The GRADE tool evaluates five main factors: risk of bias (RoB), inconsistency, indirectness, imprecision of results, and the probability of publication bias. The quality of evidence was graded on a four-point scale: “very low,” “low,” “moderate” or “high” (Balshem et al., 2011). GRADEpro (http://www.guidelinedevelopment.org/) was used to assess the level of evidence.

## 3 Results

### 3.1 Study Selection

We identified 212 studies in the six databases. After removing duplications and excluding studies that did not meet the inclusion criteria by reading the titles, abstracts, and full texts, 17 SRs (Spanier et al., 2003; Bian et al., 2006; Liu et al., 2006; Shi et al., 2008; Li et al., 2013, 2015, 2017; Zhang et al., 2014; Xiao Y. et al., 2015; Zhu et al., 2016; Dai et al., 2018; Zhou et al., 2019; Bu et al., 2020; Wang et al., 2020; Tan et al., 2020; Yao et al., 2021; Zheng et al., 2021) were found to be eligible for this overview. Among these SRs, 16 SRs and an overview of SRs (Zhang et al., 2014) were included. Two reviewers agreed to extract each SR from the overview and compare it with the SRs already searched. Thus, two non-overlapping SRs (Su et al., 2009; Huang and Zhang, 2011) were added to the overview. Finally, a total of 18 SRs (Spanier et al., 2003; Bian et al., 2006; Liu et al., 2006; Shi et al., 2008; Su et al., 2009; Huang and Zhang, 2011; Li et al., 2013, 2015, 2017; Xiao Y. et al., 2015; Zhu et al., 2016; Dai et al., 2018; Zhou et al., 2019; Bu et al., 2020; Tan et al., 2020; Wang et al., 2020; Yao et al., 2021; Zheng et al., 2021) was included in this overview (Figure 1). Six protocols (He et al., 2020; Yao et al., 2020; Han et al., 2021; Jiang et al., 2021; Lee et al., 2021; Park et al., 2021) of SR were searched during the search process; however, they were not included in this overview.

In two SRs (Spanier et al., 2003; Tan et al., 2020), we extracted only the relevant data that met the inclusion criteria. One SR (Spanier et al., 2003) conducted a study on alternative therapies, such as herbal remedies, dietary modification, digestive supplements, and psychological therapies. We included only the “herbal remedies” in this overview. The other SR (Tan et al., 2020) reported the use of HM for functional gastrointestinal disorders, including IBS, functional dyspepsia, and functional constipation. We included only the ‘IBS’ part in this overview.

### 3.2 Characteristics of Included Reviews

Among the 18 SRs included in this overview, 16 SRs conducted a meta-analysis of five to 75 RCTs; two SRs did not conduct a meta-analysis. All included SRs used either the Rome criteria I to IV, or other diagnostic criteria (e.g., Manning criteria, Chinese National criteria), for the diagnosis of IBS. A Cochrane review (Liu et al., 2006) assessed the efficacy and safety of HM compared to pharmacological interventions, placebo, or no treatment. Among the other SRs, the control intervention was classified into four categories: 1) conventional medicine (CM), 2) placebo, 3) CM or placebo, and 4) probiotics. TER was the most frequently used outcome measure in meta-analyses. TER was assessed in 15 SRs, followed by abdominal pain score in 8 SRs. The characteristics of the SRs are listed in Table 2.

**TABLE 2:** Main characteristics of included studies.

First author (Year)	Language	Studies, n (patients, n)	IBS subtype	Diagnostic criteria	Age (years)	Treatment duration	Intervention	Control	Adverse events	Main outcome measures (meta-analysis)
Spanier et al. (2003)	English	2 RCTs (NR)	NR	Rome	NR	6w, 16w	HM	CM Placebo	NR	No meta-analysis conducted
Bian et al. (2006)	English	12 RCTs (1,125)	NR	Rome I-II, Manning, TCM*	NR	10d–8w	HM HM + CM	CM	Reported	①
Liu et al. (2006)	English	75 RCTs (7,957)	D: 22 C: 5 M: 17 NR: 31	Rome I-II, Manning Chinese national conference	17–75 (mean age: 40)	9d–18w	HM HM + CM	CM Placebo HM + CM	Reported	No meta-analysis conducted
Shi et al. (2008)	English	22 RCTs (2,042)	NR	Rome I-II, TCM*	mean age: 34–49	2–18w	HM	CM Placebo	Reported	②
Su et al. (2009)	Chinese	46 RCTs (4,155)	NR	Rome Ⅰ-Ⅱ, TCM*	NR	NR	HM	CM Placebo	Reported	①
Huang and Zhang (2011)	Chinese	5 RCTs (535)	All D	Rome Ⅱ	NR	NR	HM	CM	NR	①
Li et al. (2013)	English	19 RCTs (1,510)	All C	Rome II-III, The practice of internal medicine, TCM*	NR	3–12w	HM	CM	Reported	① ③ ④
Li et al. (2015)	English	72 RCTs (6,395)	D: 29 C: 8 M: 1 NR: 34	Rome II-III, Chinese National	NR	1–12w	HM + CM	CM	Reported	①
Xiao et al. (2015a)	English	7 RCTs (954)	All D	Rome Ⅰ-Ⅲ	NR	3–16w	HM	Placebo	Reported	① ⑤ ⑥
Zhu et al. (2016)	English	15 RCTs (1,551)	All D	Rome Ⅰ-Ⅲ	NR	3–16w	HM	Placebo	Reported	① ② ⑤ ⑥ ⑦ ⑧ ⑨
Li et al. (2017)	English	11 RCTs (906)	All C	Rome II-IV	17–72	4–8w	HM	CM	Reported	① ③ ⑥ ⑩ ⑪
Dai et al. (2018)	English	23 RCTs (1,972)	All D	Rome II-IV	18–69	3–12w	HM	CM	Reported	① ② ⑥ ⑩ ⑫
Zhou et al. (2019)	English	39 RCTs (3,062)	All D	Rome	18–65	2–8w	HM	CM	Reported	① ③ ④ ⑥ ⑩ ⑬ ⑭
Bu et al. (2020)	English	47 RCTs (3,551)	D: 43 C: 3 M: 1	Rome I-IV, Manning, Kruis	18–66	2–8w	HM	Probiotics	Reported	① ③
Tan et al. (2020)	English	23 RCTs (3,338)	D: 9 C: 2 D or M: 1 NR: 11	Rome Ⅰ-Ⅲ, German IBS guidelines, Reported as IBS	mean age: 34–64	3–16w	HM	CM Placebo	Reported	① ④
Wang et al. (2020)	English	13 RCTs (868)	NR	Rome Ⅱ-Ⅲ, Consensus on diagnosis and treatment of IBS, The practice of internal medicine	NR	3-8w	HM HM + CM	CM	Reported	① ② ⑥ ⑫
Zheng et al. (2021)	English	10 RCTs (2,501)	D: 6 M: 4	Rome Ⅰ-Ⅲ	mean age: 34–64	3–16w	HM	CM Placebo	Reported	① ④ ⑥
Yao et al. (2021)	Chinese	16 RCTs (1,461)	All D	Rome Ⅲ	NR	4w	HM	CM	Reported	① ② ⑥ ⑩ ⑫ ⑬

IBS: irritable bowel syndrome, RCT: randomized controlled trial, D: diarrhea, C: constipation, M: mixed, NR: not reported, d: Day, w: Week, m: Month, HM: herbal medicine, CM: conventional medicine, AE: adverse event; TCM: Traditional Chinese medicine. Outcomes: ① Total efficacy rate ② Diarrhea score ③ Recurrence rate ④ Incidence of adverse reactions ⑤ Irritable bowel syndrome symptom severity score ⑥ Abdominal pain score ⑦ Pain threshold ⑧ Defecation threshold ⑨ Irritable bowel syndrome quality of life⑩ Frequency of defecation score ⑪ Stool form ⑫ Abdominal distention score ⑬ Fecal property score ⑭ Total symptom score *TCM criteria: diagnostic criteria published by the National Chronic Diarrhea Association of the People’s Republic of China (1986; revised 1996).

Four SRs assessed a specific single herbal prescription as an intervention: *Tong Xie Yao Fang* (TXYF) or modified TXYF in three SRs (Bian et al., 2006; Dai et al., 2018; Zhou et al., 2019) and *Shenlingbaizhu* formula in one SR (Wang et al., 2020). In the remaining SRs, two (Spanier et al., 2003) to 64 (Liu et al., 2006) kinds of herbal prescriptions were included. Of these, five SRs (Liu et al., 2006; Xiao Y. et al., 2015; Zhu et al., 2016; Wang et al., 2020; Zheng et al., 2021) described all components of each herbal prescription. Examples of herbal prescription and its components in the included SRs are presented in Supplementary Table S1.

Details of the interventions among the included SRs are summarized in Supplementary Table S2. It should be noted that duplicate contents in Supplementary Table S2 were integrated into one, hence the number of intervention and control groups was not correlated with each other.

### 3.3 Methodological Quality of Included Systematic Reviews

According to the AMSTAR 2 tool, the overall quality of one SR (Liu et al., 2006) reported by Cochrane was “moderate,” and that of one SR (Wang et al., 2020) was “low.” However, the remaining SRs had “critically low” quality. Most SRs described the research question, populations, interventions, comparators, and outcomes (PICO) of the inclusion criteria, but only five SRs preregistered the study protocol. All SRs identified the study designs for inclusion in the review, but most SRs did not search for trials or studies, grey literature, or the reference lists of the included studies. Only the Cochrane review described the list of excluded studies and their reasons. Thirteen SRs used the Cochrane handbook RoB tool for assessing the RoB of included studies. In most of the SRs that conducted a meta-analysis, appropriate methods were used for the combination of results, and all included SRs except one SR explained RoB in individual studies when discussing the results. Among the meta-analyses, except for five SRs, the remaining SRs performed adequate tests for publication bias. The details of the AMSTAR 2 result of the included SRs are shown in Table 3.

**TABLE 3:** Methodological quality assessment of the included reviews using the AMSTAR 2 tool.

Author (year)	Q1	Q2	Q3	Q4	Q5	Q6	Q7	Q8	Q9	Q10	Q11	Q12	Q13	Q14	Q15	Q16	Overall quality
Spanier et al. (2003)	N	N	Y	N	N	Y	N	PY	PY	N	NM	NM	Y	N	NM	N	Critically low
Bian et al. (2006)	N	N	Y	PY	Y	Y	N	PY	Y	N	Y	N	Y	Y	Y	N	Critically low
Liu et al. (2006)	Y	Y	Y	PY	Y	Y	Y	Y	PY	N	NM	NM	Y	N	NM	Y	Moderate
Shi et al. (2008)	Y	N	Y	PY	Y	Y	N	PY	Y	N	Y	N	Y	N	Y	N	Critically low
Su et al. (2009)	Y	N	Y	PY	N	Y	N	N	Y	N	Y	N	Y	Y	Y	N	Critically low
Huang and Zhang (2011)	Y	N	Y	PY	N	Y	N	N	Y	N	Y	N	Y	Y	Y	N	Critically low
Li et al. (2013)	Y	N	Y	PY	N	N	N	PY	Y	N	N	N	Y	Y	Y	N	Critically low
Li et al. (2015)	Y	N	Y	PY	N	Y	N	PY	Y	N	Y	N	Y	Y	Y	N	Critically low
Xiao et al. (2015a)	Y	N	Y	PY	N	Y	N	PY	Y	N	Y	Y	Y	Y	Y	Y	Critically low
Zhu et al. (2016)	Y	Y	Y	PY	Y	Y	N	PY	Y	N	Y	N	Y	Y	N	Y	Critically low
Li et al. (2017)	Y	N	Y	PY	Y	Y	N	Y	Y	N	Y	N	Y	Y	N	Y	Critically low
Dai et al. (2018)	Y	N	Y	PY	N	Y	N	PY	Y	N	N	N	Y	Y	N	Y	Critically low
Zhou et al. (2019)	Y	Y	Y	PY	Y	Y	N	PY	Y	N	Y	N	Y	N	N	Y	Critically low
Bu et al. (2020)	Y	Y	Y	PY	Y	Y	N	Y	Y	Y	Y	N	Y	Y	N	N	Critically low
Tan et al. (2020)	Y	N	Y	PY	Y	Y	N	PY	Y	N	Y	N	Y	N	Y	Y	Critically low
Wang et al. (2020)	Y	Y	Y	PY	Y	Y	N	PY	Y	Y	Y	Y	Y	Y	Y	N	Low
Zheng et al. (2021)	Y	N	Y	PY	Y	Y	N	PY	Y	N	Y	N	N	Y	Y	Y	Critically low
Yao et al. (2021)	Y	N	Y	N	N	N	N	PY	Y	N	Y	N	Y	Y	Y	N	Critically low

Q: question, Y: yes, N: no, PY: partial yes, NM: No meta-analysis was conducted.

### 3.4 Efficacy of Herbal Medicine for Irritable Bowel Syndrome

In two SRs (Spanier et al., 2003; Liu et al., 2006) that did not conduct a meta-analysis, the authors reported that HMs were effective in patients with IBS. The Cochrane review (Liu et al., 2006) included the largest number of HMs, but no meta-analysis was performed because there were no identical herbal prescriptions. The key conclusions of this study were as follows: compared with CM in 65 trials testing 51 different herbal prescriptions, 22 herbal prescriptions demonstrated a statistically significant benefit for symptom improvement, and compared with placebo, some herbal prescriptions showed a significant improvement in global symptoms. (Spanier et al., 2003) also did not conduct a meta-analysis because they included different herbal prescriptions. They included two RCTs that showed HM was significantly better than placebo or CM for symptom improvement. The details of the meta-analysis and subgroup analysis of the included SRs are shown in Supplementary Table S3.

#### 3.4.1 Total Efficacy Rate

##### 3.4.1.1 HM vs. CM

In ten SRs (Bian et al., 2006; Huang and Zhang, 2011; Li et al., 2013, 2015, 2017; Dai et al., 2018; Zhou et al., 2019; Wang et al., 2020; Yao et al., 2021; Zheng et al., 2021), HM monotherapy and adjuvant therapy with CM showed better TER results than CM. Among them, four SRs (Huang and Zhang, 2011; Dai et al., 2018; Zhou et al., 2019; Yao et al., 2021) included only patients with IBS-D and two SRs (Li et al., 2013, 2017) included only patients with IBS-C.

##### 3.4.1.2 HM vs. Placebo

In four SRs (Xiao Y. et al., 2015; Zhu et al., 2016; Tan et al., 2020; Zheng et al., 2021), HM monotherapy showed better results in terms of TER than placebo, and two SRs (Xiao Y. et al., 2015; Zhu et al., 2016) consisted of IBS-D patients.

##### 3.4.1.3 HM vs. Placebo or CM

One SR (Su et al., 2009) observed that various herbal prescriptions significantly outperformed placebo or CM in terms of TER (OR: 5.30, 95% CI: 4.38 to 6.41, *p* < 0.00001); furthermore, through subgroup analysis according to IBS subtype, IBS-D patients showed significantly better results on TER than IBS patients regardless of subtypes (OR: 5.61, 95% CI: 4.33 to 7.25, *p* < 0.00001).

##### 3.4.1.4 HM vs. Probiotics

One SR (Bu et al., 2020) reported that various herbal prescriptions showed significantly better overall symptom improvement rates than multiple types of probiotics (RR: 1.24, 95% CI: 1.18 to 1.30, *p* < 0.00001). Subgroup analysis was conducted according to different Rome criteria, duration of treatment, single- or multi-strain probiotics, and different herbal prescriptions.

#### 3.4.2 Abdominal Pain Score

##### 3.4.2.1 HM vs. CM

In five SRs (Li et al., 2017; Dai et al., 2018; Zhou et al., 2019; Wang et al., 2020; Yao et al., 2021), HM monotherapy and adjuvant therapy showed superior benefits for abdominal pain scores compared with CM monotherapy. Among them, two SRs (Dai et al., 2018; Zhou et al., 2019) included only patients with IBS-D, and one SR (Li et al., 2017) included only patients with IBS-C.

##### 3.4.2.2 HM vs. Placebo

In three SRs (Xiao Y. et al., 2015; Zhu et al., 2016; Zheng et al., 2021), HM monotherapy showed better results on abdominal pain score compared with placebo, and two SRs (Xiao Y. et al., 2015; Zhu et al., 2016) included only patients with IBS-D. Heterogeneity was low in these meta-analyses.

#### 3.4.3 Recurrence Rate

##### 3.4.3.1 HM vs. CM

In three SRs (Li et al., 2013, 2017; Zhou et al., 2019), HM showed better outcomes in terms of recurrence rate than CM monotherapy. Among them, two SRs (Li et al., 2013, 2017) included only patients with IBS-C, and one SR (Zhou et al., 2019) included only patients with IBS-D.

##### 3.4.3.2 HM vs. Probiotics

One SR (Bu et al., 2020) reported that various herbal prescriptions reduced the 1–8 months recurrence rate to 27% compared with probiotics (RR: 0.27, 95% CI: 0.18 to 0.40, *p* < 0.00001).

#### 3.4.4 Diarrhea Score

##### 3.4.4.1 HM vs. CM

In four SRs (Shi et al., 2008; Dai et al., 2018; Wang et al., 2020; Yao et al., 2021), HM monotherapy and adjuvant therapy showed better results for diarrhea scores than CM. Among these, one SR (Dai et al., 2018) included only patients with IBS-D.

##### 3.4.4.2 HM vs. Placebo

One SR (Zhu et al., 2016) reported that various herbal prescriptions showed significantly better results for the improvement of diarrhea than placebo in IBS-D patients (RR: 1.87, 95% CI: 1.60 to 2.20, *p* < 0.00001).

#### 3.4.5 Abdominal Distention Score

In three SRs (Dai et al., 2018; Wang et al., 2020; Yao et al., 2021), HM monotherapy and adjuvant therapy showed better results in terms of the abdominal distention score than CM.

#### 3.4.6 Other Outcome Measures

In four SRs (Li et al., 2017; Dai et al., 2018; Zhou et al., 2019; Yao et al., 2021) HM monotherapy and adjuvant therapy had superior results in frequency of defecation score compared with CM monotherapy. In two SRs (Xiao Y. et al., 2015; Zhu et al., 2016), HM monotherapy showed better results on the improvement of IBS-SSS score compared with placebo, and both SRs consisted of patients with IBS-D. In two SRs (Zhou et al., 2019; Yao et al., 2021) HM showed better results in improving the fecal property score as a monotherapy compared with CM, and both SRs consisted of patients with IBS-D. The results of other outcome measures, such as stool form, pain threshold, defecation threshold, and IBS-QoL scores, are reported in Supplementary Table S3.

### 3.5 Safety of Herbal Medicine for Irritable Bowel Syndrome

Except for two SRs, all the remaining SRs reported adverse events, and among them, specific adverse events were reported in 13 SRs. Across all included SRs, no serious adverse events were reported in either the HM or control groups. The details are summarized in Table 4.

**TABLE 4:** Details of adverse events among included systematic reviews.

Author (year)	Adverse events		Number of studies reporting AEs
	Intervention	Control	
Bian et al. (2006)	None		
Liu et al. (2006)	Upper gastrointestinal discomfort Headache, Nausea	Abdominal pain	17 RCTs
		Dizziness	
		Nausea	
		Mild bloating	
		Dry mouth	
		Constipation	
		Heartburn	
		Difficulty in micturition	
		Drowsiness	
Shi et al. (2008)	Distention (n = 9)	NR	NR
	Diarrhea (n = 8)		
	Abdominal pain (n = 6)		
	Constipation (n = 5)		
	Dizziness and sleepiness (n = 4)		
	Headaches (n = 4)		
	Nausea (n = 3)		
	Gastrointestinal discomfort (n = 1)		
	Upper gastrointestinal discomfort (n = 1)		
	Loss of hair (n = 1)		
	Pruritus (n = 1)		
	Paresthesia (n = 1)		
	Disturbance (n = 1)		
	Hoarseness (n = 1)		
	Shortness of breath and chest pain (n = 1)		
Su et al. (2009)	Dizziness (n = 2), Dry mouth (n = 2), Distention (n = 1), Shortness of breath and chest pain (n = 1), Skin rash (n = 1), Thyroiditis (n = 1)		13 RCTs
Li et al. (2015)	Allergic reaction, Headache, Nausea, Diarrhea, Fatigue, Loss of appetite		17 RCTs
			- No AEs: 11
			- Reporting AEs: 6
Xiao et al. (2015b)	Gastrointestinal discomfort (n = 2)	Facial nerve palsy (n = 1)	7 RCTs
	Skin rash (n = 2)		- No AEs: 4
	Thyroiditis (n = 2)		- Reporting AEs: 3
	Mild nausea (n = 2), Mild pruritus (n = 2)		
Zhu et al. (2016)	Upper gastrointestinal discomfort (n = 2)	Facial nerve palsy (n = 1)	10 RCTs- No AEs: 5- Reporting AEs: 5
	Headache (n = 2)		
	Skin rash (n = 1)		
	Thyroiditis (n = 1)		
	Flush (n = 1), Abdominal pain (n = 1), Nausea (n = 2), Pruritus (n = 2), Headache (n = 2), Low-back pain (n = 1), Dysmenorrhea (n = 1)		
Li et al. (2017)	None	Dizziness (n = 1), Dry mouth (n = 1), Low abdominal pain (n = 2), Mild diarrhea (n = 3), Bowel movements (n = 1)	2 RCTs
Dai et al. (2018)	Nausea (n = 3)	Nausea (n = 6)	12 RCTs - No AEs: 9 - Reporting AEs: 3
		Local skin rash (n = 6)	
		Abdominal discomfort (n = 2)	
		Constipation (n = 4)	
		Dry mouth (n = 1)	
Bu et al. (2020)	Headache (n = 2)	Distension (n = 5), constipation (n = 4), dry mouth (n = 2)	14 RCTs - No AEs: 12 - Reporting AEs: 2
	Low-back pain (n = 1)		
	Constipation (n = 3)		
	Dysmenorrhea (n = 1)		
	Nausea (n = 1)		
Wang et al. (2020)	Nausea	NR	5 RCTs - No AEs: 4 - Reporting AEs: 1
	Vomiting		
	Skin rashes		
Zheng et al. (2021)	Gastrointestinal disorders, Skin rash, Elevated liver enzyme		7 RCTs
Yao et al. (2021)	Nausea (n = 4)	Dry mouth (n = 4)	11 RCTs
	Dry mouth (n = 3)	Constipation (n = 4)	
	Constipation (n = 3)	Skin rash (n = 3)	
	Mild erythema (n = 1)	Nausea (n = 1)	
	Mild elevated liver enzyme (n = 1)	Abdominal pain (n = 1)	
	Mild urinary red blood cell abnormality (n = 2)	Mild elevated liver enzyme (n = 1)	
		Mild urinary red blood cell abnormality (n = 3)	

AE: adverse event, RCT: randomized controlled trial, NR: not reported.

In three SRs (Li et al., 2013; Zhou et al., 2019; Zheng et al., 2021), HM monotherapy showed a lower incidence of adverse events than CM monotherapy. (Li et al., 2013) found that various herbal prescriptions had significantly lower adverse event rates than CM in IBS-C patients (OR: 0.24, 95% CI: 0.09 to 0.65, *p* = 0.005). (Zhou et al., 2019) reported that TXYF showed a significantly lower adverse event rate than CM in patients with IBS-D (OR: 0.24, 95% CI: 0.08 to 0.86, *p* = 0.03). (Zheng et al., 2021) discovered that the proportion of adverse events was similar between various herbal prescriptions and pinaverium, but the result was not statistically significant (RR: 1.06, 95% Cl: 0.78 to 1.42, *p* = 0.79). Two SRs (Tan et al., 2020; Zheng et al., 2021) conducted a meta-analysis to compare the adverse events of HM and placebo. (Tan et al., 2020) reported that HM showed a higher adverse event rate than placebo, but the result was not statistically significant (RR: 1.40, 95% CI: 0.91 to 2.16, *p* = 0.12). (Zheng et al., 2021) discovered that various herbal prescriptions caused more adverse events than placebo (RR: 1.51, 95% Cl: 1.14 to 2.00, *p* = 0.004) (Supplementary Table S3).

### 3.6 Quality of Evidence in Included Systematic Reviews

Regarding the quality of evidence for each reported result evaluated by GRADE, “low” was the most common rating, followed by “very low,” “high,” and “moderate.” For the degrading factors, most of the meta-analyses had a high RoB and heterogeneity in results. Publication bias was not frequently observed. However, most of these results were obtained because the evaluation of publication bias for individual outcome measures was not performed in the included SRs, and a precise assessment could not be performed. All meta-analysis results revealed no factors for the upgrade. Details of the quality of evidence in the included SRs are summarized in Table 5.

**TABLE 5:** Quality of evidence in the included systematic reviews based on GRADE.

Author (year)	Outcomes (n)		Risk of bias	Inconsistency	Indirectness	Imprecision	Publication bias	Quality of evidence
Bian et al. (2006)	TER (12)		−1^a^	−1^b^	0	0	−1^c^	Very low
Shi et al. (2008)	Diarrhea (2)		−1^a^	0	0	0	−1^c^	Low
Su et al. (2009)	TER (46)		−1^a^	0	0	0	−1^c^	Low
Huang and Zhang (2011)	TER (5)		−1^a^	0	0	0	−1^c^	Low
Li et al. (2013)	TER (12)		−1^a^	0	0	0	−1^c^	Low
	RR (8)		−1^a^	0	0	0	−1^c^	Low
	AER (6)		−1^a^	0	0	0	−1^c^	Low
Li et al. (2015)	TER (72)		−1^a^	0	0	0	−1^c^	Low
Xiao et al. (2015b)	TER (6)		0	0^d^	0	0	0	High
	IBS-SSS		0	0	0	−1^e^	0	Moderate
	Abdominal pain (2)		0	0	0	0	0^f^	High
Zhu et al. (2016)	TER (7)		0	0^d^	0	0	0^f^	High
	IBS-SSS (5)		0	−2^h^	0	0	0^f^	Low
	Abdominal pain (3)		0	0	0	0	0^f^	High
	Diarrhea (4)		0	0	0	0	0^f^	High
	Pain threshold (2)		0	0	0	−1^e^	0^f^	Moderate
	Defecation threshold (2)		0	−1^b^	0	−2^e^ ^,^ ^g^	0^f^	Very low
	IBS-QoL (2)		0	0	0	−2^e^ ^,^ ^g^	0^f^	Low
Li et al. (2017)	TER (11)		−1^a^	0	0	0	−1^c^	Low
	Abdominal pain (2)		−1^a^	0	0	−1^e^	0^f^	Low
	Defecation frequency (2)		−1^a^	0	0	−1^e^	0^f^	Low
	Stool form (2)		−1^a^	0	0	−1^e^	0^f^	Low
	RR (3)		−1^a^	0	0	−1^e^	0^f^	Low
Dai et al. (2018)	TER (23)		−1^a^	0	0	0	−1^c^	Low
	Abdominal pain (14)		−1^a^	−2^h^	0	0	0^f^	Very low
	Abdominal distention (8)		−1^a^	−2^h^	0	0	0^f^	Very low
	Diarrhea (8)		−1^a^	−2^h^	0	0	0^f^	Very low
	Defecation frequency (7)		−1^a^	−2^h^	0	0	0^f^	Very low
Zhou et al. (2019)	TER (37)		0	0	0	0	0^f^	High
	Abdominal pain (11)		0	−2^h^	0	0	0^f^	Low
	Defecation frequency (6)		0	−2^h^	0	0	0^f^	Low
	Fecal property (11)		0	−2^h^	0	0	0^f^	Low
	Total symptom (8)		0	−2^h^	0	0	0^f^	Low
	AER (10)		0	0	0	0	0^f^	High
	RR (3)		0	−1^b^	0	−2^e^ ^,^ ^i^	0^f^	Very low
Bu et al. (2020)	TER (41)		−1^a^	−1^b^	0	0	−1^c^	Very low
	RR (5)		−1^a^	0	0	−1^e^	0^f^	Low
Tan et al. (2020)	TER (23)		0	−2^h^	0	0	−1^c^	Very low
	AER (12)		0	0	0	0	0^f^	High
Wang et al. (2020)	TER (8)		−1^a^	0	0	0	0	Moderate
	Diarrhea (4)		−1^a^	−2^h^	0	−1^e^	0^f^	Very low
	Abdominal pain (4)		−1^a^	−1^b^	0	−1^e^	0^f^	Very low
	Abdominal distention (3)		−1^a^	−2^h^	0	−1^e^	0^f^	Very low
Zheng et al. (2021)	Placebo	TER (8)	0	0^d^	0	0	0	High
		Abdominal pain (3)	0	0	0	0	0^f^	High
		AER (7)	0	0	0	0	0^f^	High
	CM	TER (2)	0	0	0	−1^i^	0^f^	Moderate
		AER (2)	0	0	0	−1^i^	0^f^	Moderate
Yao et al. (2021)	TER (15)		0	0	0	−1^i^	0^f^	Moderate
	Abdominal pain (10)		−1^a^	0^d^	0	0	0^f^	Moderate
	Abdominal distention (5)		−1^a^	0^d^	0	−1^e^	0^f^	Low
	Diarrhea (5)		−1^a^	−2^h^	0	0	0^f^	Very low
	Defecation frequency (4)		−1^a^	0	0	−1^e^	0^f^	Low
	Fecal property (5)		−1^a^	0^d^	0	−1^e^	0^f^	Low

TER: total efficiency rate, RR: recurrence rate, AER: adverse events rate, IBS-SSS: irritable bowel syndrome symptom severity score, IBS-QoL: Irritable bowel syndrome-quality of life, IBS-D: irritable bowel syndrome with predominant diarrhea, IBS-C: irritable bowel syndrome with predominant constipation.

a The included study (ies) had an unclear risk of selection, performance, detection, and reporting biases.

b 50 ≤ *I* ^*2*^ < 75%.

c Funnel plot indicated asymmetry.

d Sensitivity analysis was performed.

e Sample size <300.

f No publication bias assessment.

g 95 % Cl includes 0.

h *I* ^*2*^ ≥ 75.

i 95 % Cl includes 1.

## 4 Discussion

Many patients with IBS want to be treated with CAM because of unsatisfactory treatment of symptoms, reduced quality of life or the side effects of conventional treatment (Hawrelak et al., 2020). A recent study conducted in Italy reported that 45% of IBS patients diagnosed with Rome IV criteria used CAM to treat IBS (Larussa et al., 2019). Among the IBS patients receiving CAM treatment in the world, the most frequently used are HMs (43%) (Bahrami et al., 2016). According to an overview of SRs about adverse effects of HM, most of HMs (31 HMs) reported mild adverse effects (ex. pain, allergic reactions, constipation, dry mouth, etc.) associated with HMs. Moderately severe adverse effects (ex. anorexia, reversible neutropenia, coagulation abnormalities, etc.) in 15 HMs and serious adverse effects (ex. liver damage, nephrotoxicity, coma, etc.) in 4 HMs were also noted (Posadzki et al., 2013). Diverse different traditional medicines such as Korean medicine (Ko et al., 2013), traditional Chinese medicine (Zheng et al., 2021), Persian medicine (Amini-Behbahani et al., 2019), Kampo (traditional Japanese herbal medicine) (Oka et al., 2014) and Ayurveda (Tiwari et al., 2013) reported the efficacy and safety of HM in IBS. These results are important for the discovery and development of new treatments for IBS.

### 4.1 Main Findings

This overview aimed to systematically summarize the efficacy and safety of HM for IBS and assess the methodology and quality of evidence of the included SRs. Eighteen SRs were included in this overview following a comprehensive search. The included SRs showed that HM monotherapy and adjuvant therapy with CM is better for TER as a primary outcome than CM, placebo, or probiotics. Moreover, HM outperformed CM in improving individual symptoms (abdominal pain, diarrhea, abdominal distention, and frequency of defecation scores) and reducing the recurrence rate of IBS. Furthermore, HM monotherapy has a significantly lower adverse event rate than CM monotherapy, and no serious adverse events from HM interventions were reported.

IBS subtypes are classified into IBS-D, IBS-C, IBS-M, and IBS-U, according to the predominant stool pattern, and the treatment method varies accordingly (Longstreth et al., 2006). In this overview, among the 18 SRs, eight SRs (Huang and Zhang, 2011; Li et al., 2013, 2017; Xiao Y. et al., 2015; Zhu et al., 2016; Dai et al., 2018; Zhou et al., 2019; Yao et al., 2021) included specific subtypes of IBS patients, and three SRs (Su et al., 2009; Li et al., 2015; Zheng et al., 2021) conducted subgroup analysis according to IBS subtype. In IBS-D patients, HM monotherapy and adjuvant therapy showed better results in TER, individual symptoms (abdominal pain score, diarrhea score, abdominal distention score, and frequency of defecation score), recurrence rate, and IBS-SSS than CM or placebo. In patients with IBS-C, HM monotherapy was superior to CM in TER, individual symptoms (abdominal pain score and frequency of defecation score), stool form, and recurrence rate.

### 4.2 Implications for Further Research

HM has been used to treat IBS-related symptoms for centuries, and can act on multiple targets because it contains diverse components (Bi et al., 2017). HM is effective in improving IBS symptoms through suppression of visceral hypersensitivity or normalization of abnormal gastrointestinal motility (Xiao H. T. et al., 2015). Additionally, specific mechanisms of HM are related to modulation of the hypothalamus-pituitary-adrenal axis, hormones, and neurotransmitters in the enteric nervous system, intestinal microbiota, depression, anxiety, inflammation, and other factors (Xiao H. T. et al., 2015). For example, TXYF, mentioned in 10 SRs (Bian et al., 2006; Liu et al., 2006; Shi et al., 2008; Huang and Zhang, 2011; Li et al., 2015; Dai et al., 2018; Zhou et al., 2019; Bu et al., 2020; Tan et al., 2020; Yao et al., 2021) modulates intestinal motility through an inhibitory effect on colonic contraction by activating specific potassium channels and inhibiting extracellular calcium inflow (Yang et al., 2015); it also regulates inflammation by suppressing the expression of protease-activated receptor-2, which lowers the levels of IL-6 and TNF-α (Hu et al., 2013). The chemical compositions of TXYF are presented in Supplementary Table S4. Several studies have reported the efficacy of HM in treating IBS (Allescher and Abdel-Aziz, 2018; Li et al., 2019; Chen Y. et al., 2021; Yin et al., 2021), however, there are many herbal prescriptions that have not yet been studied. Therefore, more studies investigating the mechanism of action of HMs are needed in the future.

In this overview, although HM was effective in improving clinical symptoms and had a lower recurrence rate than the control interventions, the treatment duration varied from 1 to 18 weeks. Among the included SRs, two SRs (Zhou et al., 2019; Bu et al., 2020) conducted a subgroup analysis according to treatment duration. Zhou et al. (Zhou et al., 2019) conducted a subgroup analysis of abdominal pain and fecal property scores according to treatment duration (4 and 8 weeks). HM showed a significantly better abdominal pain score in the 4 weeks treatment than CM; however, in the 8 weeks treatment, the results were not statistically significant. In addition, HM outperformed CM in terms of fecal property scores in both subgroups. (Bu et al., 2020) assessed TER according to treatment duration (within 4 weeks and 4 weeks to 6 months) and found that in both subgroups, HM was better for TER compared to probiotics (Supplementary Table S3). It was difficult to propose a unified treatment duration because the treatment duration range was wide and the follow-up time differed from study to study. This may be because IBS tends to become chronic and is characterized by repeated improvement and relapse (Linedale and Andrews, 2017). Nevertheless, further research is needed on the treatment duration and recurrence rate after treatment. It may be possible to establish a more reliable HM treatment duration based on various clinical trials that consider factors such as disease severity, IBS subtype, sex, age, and response to conventional treatment.

Pattern differentiation is important in the selection of an HM. The fundamental goal of pattern-based treatment is to define the main etiology and pathophysiology in order to choose the best specific treatment strategy for each patient. To achieve optimum therapeutic benefits, different IBS patterns based on traditional medicine theory should be treated with different herbal prescriptions that are appropriate for each pattern (Chen G. et al., 2021). However, only two SR (Huang and Zhang, 2011; Xiao Y. et al., 2015) evaluated a specific pattern-based therapy: soothing the liver and strengthening the spleen (*Shugan Jianpi*) therapy. One SR (Zhu et al., 2016) conducted a subgroup analysis on TER according to the type of pattern differentiation. Accordingly, clinical research needs to be conducted in the future to determine which pattern is more effective in the use of HM for IBS. Identifying any significant differences in the application of certain pattern of IBS can help clarify the indications for HMs and can also be the basis for explaining why various HMs are effective in IBS patients.

Among the studies included in this overview, only four SRs evaluated a specific herbal prescription as an intervention, and the remaining 14 SRs assessed multiple herbal prescriptions. Five of the SRs (Liu et al., 2006; Xiao Y. et al., 2015; Zhu et al., 2016; Wang et al., 2020; Zheng et al., 2021) described all components of each herbal prescription. However, the remaining SRs did not specify the details of the components of each herbal prescription. Accordingly, it is difficult to decide which specific HM can be suggested for the treatment of IBS; thus, further SRs should be conducted on a specific HM that is, effective in treating IBS.

Moreover, the included SRs have some limitations. First, detailed evaluation criteria for outcome measurements were not present in many SRs. For example, out of 15 SRs that conducted a meta-analysis of TER, only five SRs (Li et al., 2015, 2017; Dai et al., 2018; Bu et al., 2020; Wang et al., 2020), clarified the criteria for TER. Second, the overall quality of the included SRs assessed with AMSTAR 2 was critically low. For high quality, the SRs included in this overview should have registered the protocol and described the excluded studies list. Finally, we assessed the quality of evidence, but ‘low’ was the most common among the grading outcomes. Because GRADE assessments can guide the use of these treatments for clinicians and patients in clinical practice (Dijkers, 2013), in the future, high-grade quality evidence is needed. As a result, for better evidence of HMs in IBS, well-designed SRs are required.

### 4.3 Strength of This Study

A previous study (Zhang et al., 2014) reported an overview of traditional Chinese medicine (TCM) for IBS treatment in China. It included a total of 14 SRs, of which 10 evaluated HM and four evaluated acupuncture and moxibustion, and concluded that TCM is more effective than CM in the treatment of IBS. The methodological quality of the included SRs was evaluated using the original AMSTAR tool, and the results were classified according to treatment methods (herbal prescription, powdered HM, herbal extract, acupuncture, and moxibustion). On the other hand, in this overview, we used AMSTAR 2 for assessing methodological quality. Compared to the original AMSTAR tool, AMSTAR 2 has 16 items (11 in the original), clearer response categories, an overall rating based on the weakness of critical domains, and a more thorough user guide (Shea et al., 2017). Therefore, the quality of the analysis was improved by applying a more updated tool. Furthermore, we evaluated the safety of HM and several recent SRs (published after 2014) have been added to our overview, hence the reliability of the results has increased as the analysis is based on more up-to-date clinical data. In addition, we evaluated the quality of evidence using the GRADE tool. Overall, our overview provides more expanded and standardized information on HM for IBS treatment than that of the previous overview.

### 4.4 Limitations

Despite the comprehensive search strategy was used in this overview, there is no guarantee that all relevant SRs were found. In addition, because most SRs are susceptible to publication bias (Bian et al., 2006; Shi et al., 2008; Su et al., 2009; Huang and Zhang, 2011; Li et al., 2013, 2015, 2017; Zhu et al., 2016; Bu et al., 2020; Tan et al., 2020), those biases may have been carried over into this overview.

## 5 Conclusion

The included SRs suggest that HM can be used as a single or collaborative treatment in patients with IBS. However, the quality of methodology and quality of evidence for the included SRs were generally low. Consequently, more rigorous RCTs based on pattern differentiation, specific herbal prescriptions, and IBS subtypes are required in future to better assess the safety and efficacy of HM in the treatment of IBS.

## Data Availability

The original contributions presented in the study are included in the article/Supplementary Material, further inquiries can be directed to the corresponding author.
