# Supplementary material for: An Overview of Systematic Reviews of Herbal Medicine for Irritable Bowel Syndrome
Source: Front Pharmacol. 2022 May 18;13:894122. doi: 10.3389/fphar.2022.894122 (PMC9158123; doi:10.3389/fphar.2022.894122)
Supplement: Supplementary file 1 [file Table1.DOCX]

Table S1. Examples of herbal prescription and its components in the included systematic reviews.

| **Study** | **Prescription** | **Source** | **Species, concentration** | **Quality control**  **reported?**  **(Y/N)** | **Chemical analysis**  **reported?**  **(Y/N)** |
| --- | --- | --- | --- | --- | --- |
| Spanie (2003) | Standardized herbal preparations | Mei Yu Imports, Sydney, Australia | Root of *Codonopsis pilosula* Nannf., 7g  Above ground plants of *Agastache rugosa* (Fisch. & C.A.Mey.) Kuntze*, 4.5*g  Root of *Ledebouriellae seslodis* Wolff, 3g  Seed of *Coicis lacrymal-jobi* Stapf, 7g  Root of *Bupleurum chinense* DC., 4.5g  Above ground plants of *Artemisia capillaris* Thunb*.*, 13g  Rhizome of *Atractylodis macrocephalae* Koidz, 9g  Cortex of *Mangnoliae officinalis* Rehder & E.H.Wilson, 4.5g  Peel of *Citrus reticulatae* Blanco., 3g  Rhizome of *Zingiberis offinicinalis* Roscoe, 4.5g  Cortex of *Fraxinus* *chinensis subsp. rhynchophylla* A.E.Murray, 4.5g  Sclerotium of *Poria cocos* Wolf, 4.5g  Root of *Angelicae dahuricae* Benth. & Hook.f. ex Franch*.*, 2g*,*  Seed of *Plantaginis asiatica* L., 4.5g  Cortex of *Phellodendron Chinese* C.K.Schneid., 4.5g  Root of *Glycyrrhizae uralensis* Fisch. ex DC., 4.5g  Root of *Paeoniae lactiflora* Pall., 3g  Root of *Aucklandia costus* Falc., 3g  Rhizome of *Coptis chinensis* Franch., 3g  Fruit of *Schisandra chinensis* (Turcz.) Baill*.*, 7g | N | N |
| Bian (2006) | Tongxie Yaofang | Hunan college of traditional Chinese medicine | Rhizome of *Atractylodes macrocephala* Koidz., 18g  Root of *Paeonia lactiflora* Pall., 12g  Peel of *Citrus reticulatae* Blanco., 9g  Root of *Saposhnikovia divaricata* (Turcz.) Schischk., 12g  Rhizome of *Dioscorea polystachya* Turcz., 18g  Germinated matured fruit of *Hordeum vulgare* L., 15g  Root tuber of *Curcuma wenyujin* Y.H.Chen & C.Ling, 9g  Stem bark of *Albizia julibrissin* Durazz., 12g  Root of *Glycyrrhiza uralensis* Fisch., 6g | Y – Prepared according to Chinese Pharmacopoeia | N |
| Liu (2006) | Anshen Shugan Tang | Zhejiang Wenling hospital of traditional Chinese medicine | Stem bark of *Albizia julibrissin* Durazz., 30g  Root tuber of *Fallopia multiflora* (Thunb.) Haraldson, 30g  Root of *Bupleurum chinense* DC., 10g  Root of *Paeonia lactiflora* Pall., 10g  Ripe fruit of *Citrus × aurantium* L., 10g  Sclerotium of *Macrohyporia cocos* (Schwein.) I.Johans. & Ryvarden, 10g  Above ground plants of *Portulaca oleracea* L., 30g  Root of *Raphanus raphanistrum subsp. sativus* (L.) Domin, 15g | Y – Prepared according to Chinese Pharmacopoeia | N |
| Shi (2008) | Tong Xie Yao Fang plus sini san decoction | Jiaozuo public medical hospital | Rhizome of *Atractylodes macrocephala* Koidz., 15g  Root of *Paeonia lactiflora* Pall., 15g  Peel of *Citrus reticulatae* Blanco., 15g  Root of *Saposhnikovia divaricata* (Turcz.) Schischk., 12g  Fruit of *Tetradium ruticarpum* (A.Juss.) T.G.Hartley, 12g  Fruit of *Cullen corylifolium* (L.) Medik, 30g  Seed of *Lablab purpureus* (L.) Sweet, 30g  Seed of *Plantago asiatica* L., 30g  Root of *Aucklandia costus* Falc., 12g  Ripe fruits peel of *Citrus reticulata* Blanco, 15g  Rhizome of *Coptis chinensis* Franch., 6g | Y – Prepared according to Chinese Pharmacopoeia | N |
| Su (2009) | Modified Tong Xie Yao Fang | Tianjin university of traditional Chinese medicine | Peel of *Citrus reticulatae* Blanco., 12g  Rhizome of *Atractylodes macrocephala* Koidz., 15g  Root of *Saposhnikovia divaricata* (Turcz.) Schischk., 12g  Root of *Paeonia lactiflora* Pall., 20g  Root of *Glycyrrhiza uralensis* Fisch., 9g  Rhizome of *Dioscorea polystachya* Turcz., 15g  Seed of *Lablab purpureus* (L.) Sweet, 12g  Root of *Bupleurum chinense* DC., 10g  Young fruit of *Citrus × aurantium* L., 9g  Fruit of *Amomum villosum* Lour., 9g | Y – Prepared according to Chinese Pharmacopoeia | N |
| Huang (2011) | Shugan Jianpi Zhixie Fang | Hechi people's hospital of Guangxi Zhuang Autonomous Region | Root of *Bupleurum chinense* DC., 10g  Fruit of *Amomum villosum* Lour., 10g  Rhizome of *Corydalis yanhusuo* (Y.H.Chou & Chun C.Hsu) W.T.Wang ex Z.Y.Su & C.Y.Wu, 10g  Ripe fruit of *Citrus × aurantium* L., 10g  Rhizome of *Atractylodes macrocephala* Koidz.  Rhizome of *Dioscorea polystachya* Turcz., 15g  Sclerotium of *Macrohyporia cocos* (Schwein.) I.Johans. & Ryvarden, 15g  Fruit of *Euryale ferox* Salisb., 15g  Al4(Sl4010)(0H)8·4H2O, 15g  Bark of *Magnolia officinalis* Rehder & E.H.Wilson, 20g  Root of *Glycyrrhiza uralensis* Fisch., 5g | Y – Prepared according to Chinese Pharmacopoeia | N |
| Li (2013) | Modified Piwei  Xiaoyao powder | Zhejiang Shaoxing hospital of traditional Chinese medicine | Root of *Bupleurum chinense* DC., 10g  Root tuber of *Curcuma wenyujin* Y.H.Chen & C.Ling, 10g  Fruit of *Gardenia jasminoides* J.Ellis, 12g  Root of *Paeonia lactiflora* Pall., 12g  Rhizome of *Atractylodes macrocephala* Koidz., 12g  Fruit of *Melia azedarach* L., 12g  Rhizome of *Corydalis yanhusuo* (Y.H.Chou & Chun C.Hsu) W.T.Wang ex Z.Y.Su & C.Y.Wu, 12g  Rhizome of *Pinellia ternata* (Thunb.) Makino, 10g  Bark of *Magnolia officinalis* Rehder & E.H.Wilson, 15g  Seed of *Trichosanthes kirilowii* Maxim., 12g  Ripe fruit of *Citrus × aurantium* L., 15g  Root of *Glycyrrhiza uralensis* Fisch., 10g | Y – Prepared according to Chinese Pharmacopoeia | N |
| Li (2015) | Modified Xiaoyao San | Qianxi hospital of traditional Chinese medicine | Root of *Bupleurum chinense* DC., 10g  Root of *Angelica sinensis* (Oliv.) Diels, 10g  Root of *Paeonia lactiflora* Pall., 12g  Sclerotium of *Macrohyporia cocos* (Schwein.) I.Johans. & Ryvarden, 12g  Rhizome of *Atractylodes macrocephala* Koidz., 15g  Above ground plants of *Mentha canadensis* L., 3g  Rhizome of *Zingiber officinale* Roscoe, 3 pieces  Root of *Glycyrrhiza uralensis* Fisch., 6g | Y – Prepared according to Chinese Pharmacopoeia | N |
| Xiao (2015) | Modified Tong Xie Yao Fang | The school of Chinese medicine of the Chinese university of Hong Kong | Rhizome of *Atractylodes macrocephala* Koidz., 15g  Root of *Astragalus mongholicus var. dahuricus* (DC.) Podl., 15g  Root of *Paeonia lactiflora* Pall., 15g  Rhizome of *Atractylodes lancea* (Thunb.) DC., 12g  Root of *Bupleurum chinense* DC., 9g  Peel of *Citrus reticulatae* Blanco., 9g  Root of *Saposhnikovia divaricata* (Turcz.) Schischk., 9g  Twigs of *Murraya paniculata* (L.) Jack, 9g  Peel of *Punica granatum* L., 9g  Above ground plants of *Portulaca oleracea* L., 30g  Rhizome of *Coptis chinensis* Franch., 6g | Y – Prepared according to Chinese Pharmacopoeia | N – only contamination screening was performed |
| Zhu (2016) | Modified Sishen Wan | Guangzhou Yifang Co. Ltd. | Seed of *Myristica fragrans* Houtt., 15g  Fruit of *Cullen corylifolium* (L.) Medik, 30g  Fruit of *Schisandra chinensis* (Turcz.) Baill., 9g  Fruit of *Tetradium ruticarpum* (A.Juss.) T.G.Hartley, 9g  Root of *Codonopsis pilosula* (Franch.) Nannf., 30g  Rhizome of *Atractylodes macrocephala* Koidz., 15g  Root tuber of *Curcuma wenyujin* Y.H.Chen & C.Ling, 18g  Rhizome of *Zingiber officinale* Roscoe, 10g  Fruit of *Ziziphus jujuba* Mill., 10g | Y – Prepared according to Chinese Pharmacopoeia | N |
| Li (2017) | Modified Guipi Decoction and Xiaoyaosan | Dancheng county people’s hospital | Root of *Astragalus mongholicus var. dahuricus* (DC.) Podl., 20~30g  Root of *Angelica sinensis* (Oliv.) Diels, 15~20g  Root of *Codonopsis pilosula* (Franch.) Nannf., 15~20g  Rhizome of *Atractylodes macrocephala* Koidz., 30~40g  Sclerotium of *Macrohyporia cocos* (Schwein.) I.Johans. & Ryvarden, 15~20g  Root of *Bupleurum chinense* DC., 9~12g  Root of *Paeonia lactiflora* Pall., 15~20g  Aril of *Dimocarpus longan* Lour., 12~15g  Root of *Polygala tenuifolia* Willd., 10~12g  Root of *Aucklandia costus* Falc., 5~10g  Seed of *Ziziphus jujuba* Mill., 15~20g  Root of *Bupleurum chinense* DC., 9~12g  Root of *Paeonia lactiflora* Pall., 12~15g  Above ground plants of *Mentha canadensis* L., 6~9g  Root of *Glycyrrhiza uralensis* Fisch., 6~9g | Y – Prepared according to Chinese Pharmacopoeia | N |
| Dai (2018) | Modified Tong Xie Yao Fang | Hangzhou hospital of traditional Chinese Medicine | Peel of *Citrus reticulatae* Blanco., 15g  Root of *Paeonia lactiflora* Pall., 20g  Root of *Saposhnikovia divaricata* (Turcz.) Schischk., 10g  Rhizome of *Atractylodes macrocephala* Koidz., 25g  Ripe fruit of *Citrus × aurantium* L., 10g  Root of *Bupleurum chinense* DC., 10g  Root tuber of *Curcuma wenyujin* Y.H.Chen & C.Ling, 10g  Fruit of *Melia azedarach* L., 6g | Y – Prepared according to Chinese Pharmacopoeia | N |
| Zhou (2019) | Tong Xie Yao Fang and Shenling Baizhu Powder | Lingshan county traditional Chinese medicine hospital | Root of *Paeonia lactiflora* Pall., 20g  Rhizome of *Atractylodes macrocephala* Koidz., 15g  Peel of *Citrus reticulatae* Blanco., 9g  Root of *Saposhnikovia divaricata* (Turcz.) Schischk., 6g  Sclerotium of *Macrohyporia cocos* (Schwein.) I.Johans. & Ryvarden, 30g  Seed of *Coix lacryma-jobi var. ma-yuen* (Rom.Caill.) Stapf, 20g  Seed of *Lablab purpureus* (L.) Sweet, 30g  Rhizome of *Dioscorea polystachya* Turcz., 30g  Fruit of *Amomum villosum* Lour., 6g  Seed of *Raphanus raphanistrum subsp. sativus* (L.) Domin, 10g  Stem bark of *Albizia julibrissin* Durazz., 30g  Fruit of *Citrus medica* L., 12g | Y – Prepared according to Chinese Pharmacopoeia | N |
| Bu (2020) | Tong Xie Yao Fang granule | Jiangyin Tianjiang  Pharmaceutical Co., Ltd. , Jiangsu Province, China | Rhizome of *Atractylodes macrocephala* Koidz., 15g  Root of *Saposhnikovia divaricata* (Turcz.) Schischk., 8g  Peel of *Citrus reticulatae* Blanco., 6g  Root of *Paeonia lactiflora* Pall., 12g | Y – Prepared according to Chinese Pharmacopoeia | N |
| Tan (2020) | Tong Xie Yao Fang granule | Sichuan New Green Pharmaceutical St, Sichuan, China | Rhizome of *Atractylodes macrocephala* Koidz., 10g  Root of *Saposhnikovia divaricata* (Turcz.) Schischk., 3.7g  Peel of *Citrus reticulatae* Blanco., 5g  Root of *Paeonia lactiflora* Pall., 6.7g | Y – Prepared according to Chinese Pharmacopoeia | N |
| Wang (2020) | Modified Shenlingbaizhu decoction | Beijing traditional Chinese medicine hospital affiliated to capital medical university | Root of *Codonopsis pilosula* (Franch.) Nannf., 20g  Root of *Astragalus mongholicus var. dahuricus* (DC.) Podl. 15g  Rhizome of *Atractylodes macrocephala* Koidz., 12g  Sclerotium of *Macrohyporia cocos* (Schwein.) I.Johans. & Ryvarden, 15g  Fruit of *Amomum villosum* Lour., 6g  Peel of *Citrus reticulatae* Blanco., 6g  Root of *Platycodon grandiflorus* (Jacq.) A.DC., 9g  Seed of *Lablab purpureus* (L.) Sweet, 20g  Seed of *Nelumbo nucifera* Gaertn., 15g  Seed of *Coix lacryma-jobi var. ma-yuen* (Rom.Caill.) Stapf, 30g  Root of *Glycyrrhiza uralensis* Fisch., 6g  Above ground plants of *Agastache rugosa* (Fisch. & C.A.Mey.) Kuntze, 9g | Y – Prepared according to Chinese Pharmacopoeia | N |
| Zheng (2021) | Xiang-Sha-Liu-Jun-Zi tang | Sun Ten Pharmaceutical Co. Ltd, TaiwanYao | Rhizome of *Panax ginseng* C. A. Mey., 2.5g  Rhizome of *Atractylodes macrocephala* Koidz., 5g  Sclerotium of *Macrohyporia cocos* (Schwein.) I.Johans. & Ryvarden, 5g  Root of *Glycyrrhiza uralensis* Fisch., 2g  Peel of *Citrus reticulatae* Blanco., 2g  Rhizome of *Pinellia ternata* (Thunb.) Makino, 2.5g  Fruit of *Amomum villosum* Lour., 2g  Root of *Aucklandia costus* Falc., 2g  Rhizome of *Zingiber officinale* Roscoe, 5g | Y – Prepared according to Taiwan Herbal Pharmacopoeia | Y – HPLC |
| Yao (2021) | Changning Recipe | People’s hospital of Zhongshan city | Root of *Saposhnikovia divaricata* (Turcz.) Schischk., 12g  Root of *Bupleurum chinense* DC., 10g  Root of *Paeonia lactiflora* Pall., 15g  Above ground plants of *Agrimonia pilosa* Ledeb., 20g  Rhizome of *Atractylodes macrocephala* Koidz., 10g  Sclerotium of *Macrohyporia cocos* (Schwein.) I.Johans. & Ryvarden, 15g  Root of *Platycodon grandiflorus* (Jacq.) A.DC., 6g  Ripe fruit of *Citrus × aurantium* L., 6g  Root and rhizome of *Nardostachys jatamansi* (D.Don) DC., 6g  Root of *Glycyrrhiza uralensis* Fisch., 5g | Y – Prepared according to Chinese Pharmacopoeia | N |

HPLC: High-performance liquid chromatography
