# Supplementary material for: An Overview of Systematic Reviews of Herbal Medicine for Irritable Bowel Syndrome
Source: Front Pharmacol. 2022 May 18;13:894122. doi: 10.3389/fphar.2022.894122 (PMC9158123; doi:10.3389/fphar.2022.894122)
Supplement: Supplementary file 2 [file Table2.DOCX]

Table S2. Details of interventions among the included systematic reviews.

| **Author (year)** | **Intervetion (dosage,frequency)** | **Control (dosage,frequency)** |
| --- | --- | --- |
| Spanier (2003) | ① NR(individualized or standardized) (NR,NR)  ② Ayurvedic preparation containing Aegle marmelos correa and Bacopa monniere (NR,NR) | ① Placebo (NR, NR)  ② CM or placebo (NR, NR) |
| Bian (2006) | ① Modified TXYF, TXYF (NR,NR)  ② TXYF + Xiaoling pill (NR,NR)  ③ TXYF + Nifedipinum (NR,NR) | ① Pinaverium bromide (50mg,tid)  ② Oryzandum(10mg,tid) + Dioctahedral Smectite(3g,tid)  ③ Cisapride(10mg,tid) + Lopermide(2-4mg,bid)  ④ Cisapride(10mg,tid) + Diphenoxylatum (0.2mg,tid)  ⑤ Pinaverium bromide (50mg,tid)  ⑥ Dioctahedral Smectite (3g,tid)  ⑦ Nifedipinum (10mg,tid) + Oryzandum (30mg,tid)  ⑧ Nifedipinum (10mg,tid) +Diphenoxylatum (5mg,tid)  ⑨ Nifedipinum (10mg,tid) |
| Liu^*^ (2006) | ① Individualised herbal prescription (NR,qd)  ② Yigan Fupi Tang (1 dosage daily) + Gushen Changan (3C,tid)  ③ Standard Chinese herbal formulation (5C,tid)  ④ Yichang Jian (NR,qd)  ⑤ Pingheng Zhixie decoction (NR,qd)  ⑥ Shugan Jianpi recipe (NR,qd)  ⑦ Pingyi Zhixie Tang or Pingyi Tongbian Tang (NR,qd)  ⑧ Senna leaf (6g,qd) + Fluoxetine (NR,qd) + clonazepam (1T,qd)  ⑨ Lizhong Tang (NR,qd)  ⑩ Shugan Jianpi Tang (NR,qd)  ⑪ Huanchang Tang (NR,qd)  ⑫ Gushen Changan (0.8g,tid)  ⑬ TXYF (NR,qd)  ⑭ Xiangsha Liujunzi Tang (NR,qd)  ⑮ Shuchang Wan (NR,qd)  ⑯ Fumaria officinalis (250mg,NR) or Curcuma xanthorrhiza (200mg,NR) or Ayurvedic medication (NR,NR) or a traditional spagyric remedy (NR,NR)  ⑰ Yiji Tiaochang Tang (NR,qd)  ⑱ Sanhuang Tang (NR,qd)  ⑲ TXYF (NR,qd) + Nifedipine (10mg,tid)  ⑳ Xiaoyao San (NR,qd)  ㉑ Chaimei Jiangshao Tang (NR,qd)  ㉒ Huatan Liqi Tiaofu Tang (NR,qd)  ㉓ Sijunzi Tang (NR,qd)  ㉔ Suyun Zhixie Tang (NR,bid) + (100ml for enema, qd)  ㉕ Lichang Tang (NR,qd)  ㉖ Xuanfei Tiaoqi Tang (NR,qd)  ㉗ Shugan Jianpi formula (NR,qd) + Nifedipine (10mg,tid) + Doxepin (12.5mg,tid)  ㉘ Sishen Tang (NR,qd)  ㉙ Yigan Fupi (NR,qd)  ㉚ Anshen Shugan Tang (NR,qd)  ㉛ Buzhong Yiqi Tang (NR,qd)  ㉜ Chaicang Yuxiang Tang (NR,qd)  ㉝ Jianzhong Lichang Tang (NR,qd)  ㉞ STW 5, STW 5-II (20drops,tid) or Bitter candytuft mono-extract (20drops,tid)  ㉟ Yichang San (NR,qd)  ㊱ Tibetan herbal medicine Padma Lax (2C,NR)  ㊲ Changjitai (NR,qd)  ㊳ TXYF (NR,qd)+ Clostridium butyricum(2C,tid)  ㊴ Huoxiang Zhengqi capsule (6g,tid)  ㊵ Sanbai San (NR,qd)  ㊶ Yigan Fupi Huatan Quyu (NR,qd)  ㊷ Shugan Jianpi Fang (NR,qd)  ㊸ Geqinshu Jiangshuocao Tang (NR,qd)  ㊹ Tiaoli Ganpi recipe (NR,qd) + oryzanol (30mg,tid)  ㊺ Tiaogan Yichang Tang (NR,qd)  ㊻ Pinggan Jianpi (NR,qd)  ㊼ Chaihu Shugan Yin (NR,qd)  ㊽ Ayurvedic preparation (6g,tid)  ㊾ Tiaogan Shipi recipe (NR,qd)  ㊿ Shugan Lipi recipe (NR,qd) + VitaminB1 (20mg,tid) + oryzanol (30mg,tid)  Xianshi capsule (4C,tid)  Changji Fang (NR,qd) + Phenobarbital (15mg,tid) + belladona (10ml,tid) + Smecta (3g,tid)  Liqi Anchang Tang (NR,qd) + Jiechang Ning for enema (NR,qd)  Jianpi Shugan Tang (NR,qd)  Baile Ercha (5C,tid)  Ganpi Lunzhi (NR,qd)  Jiechang Kang (4-6T,tid)  Sugan Renchang recipe (NR,qd)  Shenling Baishu San (NR,qd)  Xuefu Zhuyu Tang (NR,qd)  Shunji Heji (25ml,tid)  Banxia Xiexin Tang (NR,qd)  Buzhong Yiqi Tang modified (NR,qd)  Zhongyao Heji (50ml,bid) | ① Oryzanol (NR,NR) + Nifedipine(NR,NR) Cisapride (NR,NR) or Indomethacin(NR,NR) + Lacidophilin(NR,NR)  ② Pinaverium bromide (50mg,tid)  ③ Pinaverium bromide (50mg,tid) + Smecta(1pk,tid)  ④ Nifedipine (5mg,tid) + Live bifidobacterium  ⑤ Cisapride (10mg,tid) (5mg,tid)  ⑥ Cisapride (5mg,tid) + Fluoxetine (NR,qd) + Clonazepam (1T,qd)  ⑦ Sodium cromoglicate (200mg,tid) + Diazepam (2.5mg,tid) + Vitamin B1 (100mg,tid)  ⑧ Nifedipine (10mg,tid) + Oryzanol (30mg,tid) + Berberine (300mg,tid)  ⑨ Anisodamine (10mg,bid) + Oryzanol (20mg,tid)  ⑩ Nifedipine (10mg,tid) + Bifidobacterium (2C,bid)  ⑪ Gushen Changan (0.4g,tid) + Oryzanol (30mg,tid)  ⑫ Diazepam, propantheline, domperidone (NR,NR)  ⑬ Cisapride (10mg,tid)  ⑭ Nifedipine (5mg,tid) + Oryzanol (20mg,tid)  ⑮ Doxepin (25mg,tid) + Nifedipine (10mg,tid)  ⑯ Furazolidone (100mg,tid) + Retardin (2T,bid)  ⑰ Nifedipine (10mg,tid)  ⑱ Oryzanol (20mg,tid) + Loperamide (20mg,tid)  ⑲ Nifedipine (10mg,tid) + Oryzanol (20mg,tid) + Doxepin (12.5mg/25mg,bid)  ⑳ Smecta (3g,tid)  ㉑ VitaminB1 (20mg,tid) + Oryzanol (20mg,tid) (30mg,tid)  ㉒ Berberine (0.3g,tid), Retardin (2T,tid), Chlorpheniramine (NR,tid) + Gentamycin 240,000units, Metronidazole 1g, in 100 mL of 0.9% of NaCl for enema every night  ㉓ Licheiformobiogen (500mg,tid) + Lacidophilin (1.2g,tid)  ㉔ Cisapride (5mg,tid) + Oryzanol (30mg,tid)  ㉕ Nifedipine (10mg,tid) + Doxepin (12.5mg,tid)  ㉖ Mebevenine (50mg,tid)  ㉗ Domperidone (10mg,tid) + Nifedipine (10mg,tid) + Oryzanol (10mg,tid)  ㉘ Oryzanol (40mg,tid) + Sodium cromoglicate (500mg,tid)  ㉙ Oryzanol (20mg,tid) (30mg,tid) (50mg,tid)  ㉚ Oryzanol (NR,NR) (10mg,tid) + Berberine (NR,NR) (300mg,tid)  ㉛ Retardin (200mg,tid) + Cisapride (10mg,tid)  ㉜ Clostridium butyricum (2C,tid)  ㉝ Anisodamine (10mg,tid)  ㉞ Oryzanol (20mg,tid) (30mg,tid) (60mg,tid) + Nifedipine (10mg,tid)  ㉟ Retardin (2T,tid) (2T,bid)  ㊱ Smecta (3g,tid) + VitaminB1 (100mg,tid)  ㊲ Gentamycin (80,000U,qd) + Berberine (300mg,qd)  ㊳ Oryzanol (50mg,tid) + Nifedipine (100mg,tid) + Bifico(triple viable biogen) (3T,tid)  ㊴ Clidinium bromide, Chlordiazepoxide and Isaphaghulla (6g,tid)  ㊵ Phenobarbital (15mg,tid) + Belladona (10ml,tid) + Smecta (3g,tid)  ㊶ Nifedipine (10mg,tid) + Hydrocortecoid 100 mg in 200 mL of warmed water for enema  ㊷ Diazepam (2.5mg,tid) + Propantheline (15mg,tid)  ㊸ SMZ-TMP-co (1g,tid), Propantheline (30mg,tid), Oryzanol (20mg,tid), Chlordiazepoxide (20mg,tid), Subcarbonate (0.6g,tid) + 100mL of 3% berberine plus 20mL Novocaine for enema (qd)  ㊹ Licheiformobiogen (0.5g,tid)  ㊺ Loperamide (2mg,qd)  ㊻ Sulfasalazine (0.5g,tid) + Retardin (5mg,tid) + Anisodamine (5mg,tid)  ㊼ Colloidal bismuth tartrate (165mg,tid)  ㊽ Live Bifidobacterium (2C,bid)  ㊾ Placebo (5C,tid) (NR,NR) (20drops,tid) (2C per day) (6g,tid) (2g,tid) |
| Shi (2008) | ① Lizhong Huoxie decoction (NR,NR)  ② Individualized herbal prescription (NR,NR)  ③ Modified TXYF (NR,NR)  ④ Huatan Liqi Tiaofu decoction (NR,NR)  ⑤ Geqin Shujiang Shaocao decoction (NR,NR)  ⑥ Xianshi Capsule (NR,NR)  ⑦ Huanchang decoction (NR,NR)  ⑧ Congpi Lunzhi Formula (NR,NR)  ⑨ Xiangsha Liujunzi decoction (NR,NR)  ⑩ Shunji mixture (NR,NR)  ⑪ Padma Lax (Tibetan herbal formula) (NR,NR)  ⑫ Changkang Capsule (NR,NR)  ⑬ Wuma Simo decoction (NR,NR)  ⑭ Gegan Qinlian Pellet (NR,NR)  ⑮ Changning Yin decoction (NR,NR)  ⑯ Changjitai decoction (NR,NR)  ⑰ Liyiting decoction (NR,NR)  ⑱ TXYF + Sini san decoction (NR,NR)  ⑲ Tongxie yihao capsule (NR,NR)  ⑳ STW5, STW5-Ⅱ or Bitter Candytuft (NR,NR)  ㉑ Jiejing Yiji decoction (NR,NR)  ㉒ Curcama or Fumitory (NR,NR) | ① Oryzanol, Nifedipine (NR,NR)  ② Placebo (NR,NR)  ③ Salazosulfapyridine, Diphenoxylate, Anisodamine, Amitriptyline, Placebo (NR,NR)  ④ Smecta (NR,NR)  ⑤ Smecta, vitB (NR,NR)  ⑥ Smecta, Dicetel (NR,NR)  ⑦ Anisodamine, Oryzanol (NR,NR)  ⑧ Bacillus Licheniformis (NR,NR)  ⑨ Diazepam, Propantheline, Domperidone (NR,NR)  ⑩ Bitinal (NR,NR)  ⑪ Amitriptyline (NR,NR)  ⑫ Cisapride (NR,NR)  ⑬ Nifedipine (NR,NR)  ⑭ Diphenoxylate (NR,NR)  ⑮ Dicetel (NR,NR)  ⑯ Salazosulfapyridine, Diphenoxylate, Anisodamine, Amitriptyline, Placebo (NR,NR)  ⑰ Dicetel, Domperidone, Loperamide, Doxepin (NR,NR)  ⑱ Cerekinon (NR,NR) |
| Su (2009) | NR | NR |
| Huang (2011) | ① Modified TXYF (NR,NR)  ② Shugan Jianpi Huashi Fang (NR,NR)  ③ Tongxieanshu Tang (NR,NR)  ④ Shugan Jjianpi Zhixie Fang (NR,NR)  ⑤ Xiaoyao Tongxie Yin (NR,NR) | ① Dicetel (NR,NR)  ② 654-2 + Diphenoxylate + Trimebutine (NR,NR)  ③ Nifedipine + Oryzanol + Vitamin B1 + Loperamide (NR,NR)  ④ Nifedipine + Oryzanol + Loperamide (NR,NR) |
| Li (2013) | ① Self-prescribedrecipe (NR,qd) (NR,NR)  ② Yiji-II (NR,qd)  ③ Modified Jieyu Runchang soup (NR,qd)  ④ Modified Piwei Xiaoyao powder (NR,qd)  ⑤ Sini powder + Wuren soup (NR,qd)  ⑥ Jianpi Daozhi soup (NR,qd)  ⑦ Modified recipe of Shugan Runchang (NR,qd)  ⑧ Modified Sinisan (NR,qd) (NR,bid)  ⑨ Self-prescribed Huanji Tongbian soup (NR,NR)  ⑩ Yimu Hezhong soup (NR,qd)  ⑪ Modified Simo soup + Xiaoyao powder (NR,qd)  ⑫ Self-prescribed Tongbian soup (NR,NR) (200ml,bid)  ⑬ Modified Erhe soup (NR,qd) | ① Cisapride (5mg,tid) (10mg,tid)  ② Polyethyleneglycol (20g,qd) (10g,bid)  ③ Trimebutine (100mg,tid) (200mg,tid)  ④ Tegaserod (6mg,bid)  ⑤ Cisapride (10mg,tid) + Doxepin (25mg,tid)  ⑥ Mosapride (5mg,tid) |
| Li (2015) | ① Liuwei Anxiao casple (NR,NR)  ② TXYF, modified TXYF decoction (NR,NR)  ③ Individualized herbal prescription formula (NR,NR)  ④ Simo Tang oral solution (NR,NR)  ⑤ Jianpi Liqi decoction (NR,NR)  ⑥ Runchangshu modified decoction (NR,NR)  ⑦ Maziren decoction (NR,NR)  ⑧ Tiaohe Ganpi recipe (NR,NR)  ⑨ Anshen Ningchang modified decoction (NR,NR)  ⑩ Gushen Changan capsule (NR,NR)  ⑪ Addition Sini San (NR,NR)  ⑫ TCM syndrome differentiation therapy (NR,NR)  ⑬ Xiaoyao San decoction, Modified Xiaoyao San (NR,NR)  ⑭ Jianpi Tiaochang decoction (NR,NR)  ⑮ Liuwei Muxiang capsule (NR,NR)  ⑯ Modified Liujunzi decoction (NR,NR)  ⑰ Shugan Liqi and Jianpi Huashi or Runchang Tongbian recipe (NR,NR)  ⑱ Modified TXYF + Sini San decoction (NR,NR)  ⑲ Modified Shenling Baizhu San (NR,NR)  ⑳ Wuling San + modified TXYF (NR,NR)  ㉑ Zhenren Yiangzang decoction + Shengma Gegen decoction (NR,NR)  ㉒ SLBZ granules, SLBZ decoction (NR,NR)  ㉓ Modified TXYF + SLBZ decoction (NR,NR)  ㉔ Tiaogan Fupi decoction (NR,NR)  ㉕ Shugan Yipi decoction (NR,NR)  ㉖ Xiaojian Zhong decoction (NR,NR)  ㉗ Runchang Shu recipe (NR,NR)  ㉘ Shugan Jianpi decoction (NR,NR)  ㉙ Yiji Ning decoction (NR,NR)  ㉚ Modified Mazi Renwan (NR,NR)  ㉛ Shugan Liqi Jianpi dedoction (NR,NR)  ㉜ Shgan Jianpi recipe (NR,NR)  ㉝ Yigan Fupi decoction (NR,NR)  ㉞ Chaihu Shugan San decoction (NR,NR)  ㉟ Sishen Wan modified decoction (NR,NR)  ㊱ Zhizhu Kuanzhong capsule (NR,NR)  ㊲ Shugan Jieyu capsule (NR,NR)  ㊳ Zhenqi Fuzheng powder (NR,NR) | ① Mosapride, Oryzanol, Vitamin B1 (NR,NR)  ② Trimebutine, Bifidobacterium (NR,NR)  ③ Mosapride (NR,NR)  ④ Mosapride, Bifidobacterium (NR,NR)  ⑤ Trimebutine (NR,NR)  ⑥ Cisapride, Vitamin B1, Vitamin B6, Vitamin C (NR,NR)  ⑦ Oryzanol, Doxepin, Cisapride, Diphenoxylate (NR,NR)  ⑧ Pinaverium bromide, Loperamide, Domperidone, Antidepressant (NR,NR)  ⑨ Alverine (NR,NR)  ⑩ Bifidobacterium (NR,NR)  ⑪ Pinaverium bromide (NR,NR)  ⑫ Flupentixol + Melitracen (NR,NR)  ⑬ Symptomic drugs (NR,NR)  ⑭ Bifidobiogen (NR,NR)  ⑮ Itopride, Oryzanol (NR,NR)  ⑯ Diphenoxylate, Colloidal bismuth, Cisapride (NR,NR)  ⑰ Nifedipine, Oryzanol (NR,NR)  ⑱ Bifidobacterium, Pinaverium bromide, Polydluosan (NR,NR)  ⑲ Smecta, Bifidobacterium (NR,NR)  ⑳ Pinaverium bromide, Phenolpthalein (NR,NR)  ㉑ Nifedipine, Trimebutine (NR,NR)  ㉒ Pinaverium bromide, Smecta (NR,NR)  ㉓ Pinaverium bromide, Oryzanol (NR,NR)  ㉔ Sucralfate, Pinaverium bromide, Loperamide, Domperidone (NR,NR)  ㉕ Smecta (NR,NR)  ㉖ Anisodamine, Diphenoxylate, Bifidobacterium (NR,NR)  ㉗ Doxepin, Polyzym (NR,NR)  ㉘ Berberine, Smecta, Oryzanol (NR,NR)  ㉙ Trimebutine, Flupentixol + Melitracen (NR,NR)  ㉚ Trimebutine, Clostridium butyricum (NR,NR)  ㉛ Trimebutine, Bifidobacterium, Pancreatin Enteric-coated capsule (NR,NR)  ㉜ Pinaverium bromide, symptomatic drugs (NR,NR)  ㉝ Pinaverium bromide, Loperamide (NR,NR)  ㉞ Trimebutine, Oryzanol (NR,NR)  ㉟ Berberine (NR,NR)  ㊱ Bifidobacterium, Pinaverium bromide (NR,NR) |
| Xiao^*^ (2015) | ① Standard formula (ingredients detail shown) (5C,tid)  ② Traditional Chinese herbal formula (1Pk,bid)  ③ Chang Ji Tai (1Pk,bid)  ④ Tong Xie Ning granule (5g,tid)  ⑤ Chang An Yi Hao decoction (150mL,tid)  ⑥ ShuGan JianPi WenShen decoction (150mL,tid)  ⑦ Chang Ji Tai granule (1Pk,bid) | ① Placebo (NR,NR) |
| Zhu^*^  (2016) | No specific formula name, only the ingredients of each formula + TCM therapy (ex. Soothing the liver and invigorating the spleen) was presented (NR,NR) | ① Placebo (NR,NR) |
| Li (2017) | ① Decoction of liver -soothing and stomach -regulating (200mL,bid)  ② Yiji II recipe (NR,tid)  ③ Decoction of Buqi Jianpi (150mL,bid)  ④ Yangxue Tongfu Decoction (300mL,bid)  ⑤ Compound granules with Regulating Qi and Moistening the Intestines (100mL,bid)  ⑥ Modified Sini Powder and Liumo Decoction (NR,bid)  ⑦ Modified Fluid-increasing Decoction (NR,bid)  ⑧ Decoction of Replenishing Qi to invigorate the Spleen and ventilating the Lung (200mL,bid)  ⑨ Modified Guipi Decoction + Xiaoyao Powder (NR,bid)  ⑩ Modified Sanzang Tiaohe Runchang Decoction (150mL,bid)  ⑪ Treatment base on TCM Syndrome Differentiation (NR,bid) | ① Cisapride tablets (5mg,tid)  ② Mosapride citrate tablets (5mg,tid) |
| Dai (2018) | ① Modified TXYF (NR,NR) | ① Montmorillonite powder + Pinaverium bromide tablets (NR,NR)  ② Pinaverium bromide tablets (NR,NR)  ③ Montmorillonite powder + Trimebutine maleate (NR,NR)  ④ Bacillus licheniformis + Pinaverium bromide tablets (NR,NR)  ⑤ Montmorillonite powder (NR,NR)  ⑥ Loperamide (NR,NR)  ⑦ Miyarisam (NR,NR)  ⑧ Glutamine compound enteric capsule (NR,NR)  ⑨ Pinaverium bromide tablets + Live Combined bifidobacterium + lactobacillus and enterococcus Powder (NR,NR) |
| Zhou (2019) | ① TXYF (NR,NR) | ① Dioctahedral smectite + Pinaverium bromide (NR,NR)  ② Pinaverium bromide + Oryzanol (NR,NR)  ③ Pinaverium bromide (NR,NR)  ④ Trimebutine + Bifid Triple Viable Capsules (NR,NR)  ⑤ Trimebutine (NR,NR)  ⑥ Pinaverium bromide + Bifid Triple Viable Capsules (NR,NR)  ⑦ Bifidobacterium tetra viable tablets (NR,NR)  ⑧ Bacillus subtilis and Enterococcus bacteria capsule (NR,NR)  ⑨ Bifid triple viable capsules (NR,NR)  ⑩ Montmorillonite + Bacillussubtilis and Enterococcus bacteria capsule (NR,NR)  ⑪ Combined Bifidobacterium and Lactobacillus tablets (NR,NR)  ⑫ Loperamide (NR,NR)  ⑬ Otilonium bromide (NR,NR)  ⑭ Dioctahedral smectite (NR,NR)  ⑮ Trimebutine + Bifid triple viable capsules +Vitamin K (NR,NR)  ⑯ NR  ⑰ Bifidobiogen (NR,NR)  ⑱ Dioctahedral smectite (NR,NR)  ⑲ Oryzanol + Dioctahedral smectite (NR,NR)  ⑳ Bifidobacterium tetra viable tablets + Pinaverium bromide (NR,NR) |
| Bu (2020) | ① Modified SLBZ + Sishen Wan (100-150mL,NR)  ② Huangqi Guizhi Wuwu Tang (150mL,NR)  ③ Tiaochang Fang (NR,NR)  ④ Modified Shenling Baishu San (150mL,NR)  ⑤ Modified Buhuanjin Zhengqi San (200mL,NR)  ⑥ Modified Wenshen Jianpi Fang (NR,NR)  ⑦ Wangshi Yigan Fupi Tang (NR,NR)  ⑧ TXYF, Modified TXYF (NR,NR) (150mL,NR)  ⑨ Modified Qiwei Baizhu San + TXYF (75mL,NR)  ⑩ Tiaogan Zhixie Tang (NR,NR)  ⑪ Huoxiang Zhengqi San (1package,NR)  ⑫ TXYF + SLBZ (NR,NR)  ⑬ Modified Buhuanjin Zhengqi San (200mL,NR)  ⑭ Modified TXYF + Banxia Xiexin Tang (6g,NR)  ⑮ Shugan Jianpi Qufeng Tang (NR,NR)  ⑯ Jianpi Huashi Fang (100mL,NR)  ⑰ Modified Lizhong Tang (NR,NR)  ⑱ Shuxie Fang (200mL,NR)  ⑲ Changping Tang (100mL,NR)  ⑳ Simo Tang Koufuye (20mL,NR)  ㉑ Jianpi Huazhuo Tongluo Fang (100mL,NR)  ㉒ Modified Qiwei Baizhu San (NR,NR)  ㉓ Modified Chaishao Liujun Keli (150mL,NR)  ㉔ Modified Dachaihu Tang (100mL,NR)  ㉕ Modified Fuzi Lizhong Tang + Sishen Wan (75mL,NR)  ㉖ Wenzhong Zhixie Tang (150mL,NR)  ㉗ Heganpi Yin (250mL,NR)  ㉘ Pinggan Zhixie Tang (NR,NR)  ㉙ Jianpi Huazhi Wan (10g,NR)  ㉚ Modified Sishen Wan (100mL,NR)  ㉛ Erzhu Tang (NR,NR)  ㉜ Modified Sini San + Lizhong Tang (150mL,NR)  ㉝ Modified Jijiao Lihuang Tang (NR,NR)  ㉞ Jianpi Huashi Fang (100mL,NR)  ㉟ Tongxie Sishen Tang (NR,NR)  ㊱ Shenling Guchang Keli (150-200mL,NR)  ㊲ Ginger root powder (1g,NR)  ㊳ Shenqu (100mg,NR) | ① Triple Bifidobacterium preparations (200mg,tid) (400mg,bid) (420-630mg,bid-tid) (420mg,tid) (210mg,bid) (420mg,bid) (630mg,bid) (840mg,bid)  ② Bacillus Licheniformis preparations (500mg,tid)  ③ Triple Lactobacillus preparations (660mg,tid)  ④ Mixture preparations of Bacillus Subtilis and Enterococcus Faecium (500mg,tid) (500mg,bid)  ⑤ Clostridium Butyricum preparations (1-2capsule,tid) (700mg,bid)  ⑥ mixture preparations of 7 probiotics (1capsule,tid)  ⑦ Bifidobacterium adolescentis preparations (700mg,bid) (700mg,tid) (2capsules,tid) (50mg,tid)  ⑧ Mixture preparations of Clostridium (3capsules,bid)  ⑨ Quadruple Bifidobacterium infantis preparations (1500mg,tid)  ⑩ Brewer’s yeast tablets (500mg,qd) |
| Tan (2020) | ① A Chinese Herbal Medicine (NR,NR)  ② Crofelemer (latex of Croton lechleri) (NR,NR)  ③ TXYF (NR,NR)  ④ IQP-CL-101 (NR,NR)  ⑤ Peppermint oil (NR,NR)  ⑥ Aloe vera (NR,NR)  ⑦ Enteric coated capsules of anise oil (NR,NR)  ⑧ Ginger (NR,NR)  ⑨ Curcumin and Fennel Essential Oil (NR,NR)  ⑩ Carmint (NR,NR)  ⑪ Ayurvedic herbs (NR,NR)  ⑫ Padma Lax (Tibetan herbal formula) (NR,NR)  ⑬ Gwakhyangjeonggisan (NR,NR)  ⑭ Aloe barbadensis Mill. Extract (NR,NR)  ⑮ Modified Sishen Wan (NR,NR)  ⑯ Chang’ an I Recipe (NR,NR)  ⑰ Tong-xie-ning (NR,NR)  ⑱ Tongxie formula (Personalized Formula) (NR,NR)  ⑲ St John's Wort (NR,NR) | ① Placebo (NR,NR)  ② Pinaverium bromide (NR,NR) |
| Wang^*^ (2020) | ① SLBZ (6g,bid) (NR,bid)  ② SLBZ + pinaverium bromide (150mL,bid)  ③ SLBZ +Trimebutine Maleate (6g,bid) (150mL,bid)  ④ SLBZ + bifidobacteria (NR,bid)  ⑤ SLBZ + pinaverium bromide + oryzanol (NR,bid) | ① Dioctahedral smectite (3g,tid)  ② Pinaverium bromide (50mg,tid)  ③ Trimebutine Maleate (100mg,tid)  ④ Bifidobacteria (630mg,bid) (420mg,bid)  ⑤ Bifico (NR,tid)  ⑥ Pinaverium bromide (50mg,tid) + Oryzanol (10mg,tid)  ⑦ Lactic acid bacteria (2pills,bid) + Pinaverium bromide (50mg,tid) |
| Zheng^*^ (2021) | ① Standardized or individualized herbal prescription (5C,tid)  ② Standardized herbal prescription (1Pk,tid) (1Pk,bid) (6C,tid) (400mL,tid) (3g,tid) (5g,tid) (NR,bid) | ① Pinaverium bromide (NR,NR)  ② Pinaverium bromide, Placebo (NR,NR)  ③ Placebo (NR,NR) |
| Yao (2021) | ① Modified Jiawei Shipi San (NR,NR)  ② TXYF + Chaishao Liujunzi Tang (NR,NR)  ③ Doubled Changbing Fang (NR,NR)  ④ Chang Ning Fang (NR,NR)  ⑤ Fuzi Lizhong Tang (NR,NR)  ⑥ Modified Yi Gong San (NR,NR)  ⑦ Shugan Jianpi Fang (NR,NR)  ⑧ Xinjia TXYF (NR,NR)  ⑨ Differential treatment herbal prescription (NR,NR)  ⑩ TXYF (NR,NR)  ⑪ Xifeng Huashi Fang (NR,NR)  ⑫ TXYF + Xiangsha Liujunzi (NR,NR)  ⑬ Chang Xie Ning (NR,NR)  ⑭ Yiqi Guchang Fang (NR,NR)  ⑮ Doubled modified Li Chang Yin (NR,NR)  Tongxiening Granules (NR,NR) | ① Pinaverium bromide (50mg,tid) |

Duplicate contents were integrated into one, so the number of intervention and control groups was not correlated with each other. ^*^Describe all components of each herbal prescription. C: Capsule, T: Tablet, Pk: Pack, qd: Quaque die (=once a day), bid: Bis in did (=two times a day), tid: Ter in die (=three times a day), TXYF: Tongxieyaofang, NR: Not reported, SLBZ: Shenlingbaizhu formula
