# Supplementary material for: An Overview of Systematic Reviews of Herbal Medicine for Irritable Bowel Syndrome
Source: Front Pharmacol. 2022 May 18;13:894122. doi: 10.3389/fphar.2022.894122 (PMC9158123; doi:10.3389/fphar.2022.894122)
Supplement: Supplementary file 3 [file Table3.DOCX]

Table S3. Meta-analysis results of the included systematic reviews.

| **Comparison** | **Author (year)** | **RCTs, n**  **(patients, n)** | **Meta-analysis results** | **Subgroup analysis** |
| --- | --- | --- | --- | --- |
| Total efficacy rate | | | | |
| HM  (or HM+CM)  vs  CM | Bian (2006) | 12 RCTs, n=1,124 | RR 1.35, 95% Cl 1.21 to 1.50,  p<0.00001, *I*²=53.3% | Follow-up period  Near-term efficacy: 4 RCTs, n=285; RR 1.34, 95% Cl 1.16 to 1.54, p < 0.0001, *I*²=0%  Short-term efficacy: 2 RCTs, n=228; RR 1.39, 95% Cl 1.17 to 1.64, p = 0.0001, *I*²=7.4%  Long-term efficacy: 6 RCTs, n=611; RR 1.34, 95% Cl 1.12 to 1.61, p = 0.002, *I*²=73.3% |
|  | Huang (2011) | 5 RCTs, n=535 | OR 6.95, 95% Cl 4.09 to 11.79, p<0.00001,  χ^2^=3.74 (p=0.44) | NR |
|  | Li (2013) | 12 RCTs, n=1,057 | OR 2.61, 95% Cl 1.93 to 3.52, p<0.00001, *I*²=26% | NR |
|  | Li (2015) | 72 RCTs, n=6,395 | RR 1.21, 95% Cl 1.18 to 1.24, p<0.00001, *I*²=7% | IBS subtype  IBS-D: 29 RCTs, n=2,525; RR 1.19, 95% Cl 1.15 to 1.24, p<0.00001, *I*²=15%  IBS-C: 8 RCTs, n=753; RR 1.23, 95% Cl 1.13 to 1.33, p<0.00001, *I*²=32%  Not specify the type: 34 RCTs, n=3,037; RR 1.22, 95% Cl 1.18 to 1.27, p<0.00001, *I*²=17% |
|  | Li (2017) | 11 RCTs, n=906 | OR 4.00, 95% Cl 2.74 to 5.84, p<0.00001, *I*²=0% | NR |
|  | Dai (2018) | 23 RCTs, n=1,972 | OR 4.04, 95% Cl 3.09 to 5.27, p<0.00001, *I*²=0% | Types of drugs in the control group  Pinaverium bromide tablets: 15 RCTs, n=1,322; OR 4.14, 95% Cl 2.98 to 5.75, p<0.00001, *I*²=0%  Pinaverium bromide tablets + Other CM: 3 RCTs, n=248; OR 4.14, 95% Cl 1.67 to 10.07, p=0.002, *I*²=0%  Other CM: 5 RCTs, n=376; OR 3.73, 95% Cl 2.20 to 6.34, p<0.00001, *I*²=34% |
|  | Zhou (2019) | 37 RCTs, n=2,922 | OR 4.61, 95% Cl 3.67 to 5.78, p<0.00001, *I*²=0% | NR |
|  | Wang (2020) | 8 RCTs, n=487 | OR 2.38, 95% Cl 1.43 to 3.95, p=0.0008, *I*²=0% | Criteria of cure rate  cure rate1^*^: 6 RCTs, n=369; OR 2.49, 95% Cl 1.38 to 4.48, p=0.002, *I*²=0%  cure rate2^†^: 2 RCTs, n=118; OR 2.10, 95% Cl 0.77 to 5.71, p=0.15, *I*²=0% |
|  | Zheng  (2021) | 2 RCTs, n=516 | RR 1.10, 95% Cl 0.99 to 1.22, p=0.16, *I²*=49% | NR |
|  | Yao (2021) | 15 RCTs, n=1,119 | RR 1.25, 95% Cl 1.18 to 1.33, p<0.00001, *I²*=0% | NR |
| HM  vs  Placebo | Xiao (2015) | 6 RCTs, n=917 | RR 1.61, 95% Cl 1.24 to 2.10, p=0.0004, *I*²=70.0% | NR |
|  | Zhu (2016) | 7 RCTs, n=815 | RR 1.62, 95% Cl 1.31 to 2.00, p<0.00001, *I*²=59% | Types of pattern differentiation  WKIS therapy: 2 RCTs, n=320; RR 2.06, 95% Cl 1.71 to 2.47, p<0.00001, *I*²=0%  SLIS therapy: 5 RCTs, n=440; RR 1.42, 95% Cl 1.08 to 1.86, p=0.01, *I*²=57% |
|  | Tan (2020) | 23 RCTs, n=3,338 | RR 1.62, 95% Cl 1.32 to 1.97, p<0.00001, *I*²=78% | Diagnosis criteria  Rome Ⅰ: 2 RCTs, n=196; RR 3.36, 95% Cl 0.84 to 13.44, p=0.09, *I*²=81%  Rome Ⅱ: 7 RCTs, n=483; RR 1.44, 95% Cl 0.88 to 2.36, p=0.15, *I*²=76%  Rome Ⅲ: 13 RCTs, n=2,180; RR 1.60, 95% Cl 1.26 to 2.04, p=0.0001, *I*²=81%  non-Rome: 1 RCTs, n=62; RR 1.84, 95% Cl 1.29 to 2.62, p=0.0007 |
|  | Zheng  (2021) | 8 RCTs, n=1,716 | RR 5.30, 95% Cl 1.33 to 2.33, p<0.001, *I²*=81.1% | IBS subtypes  IBS-D: 6 RCTs, n=1,475; RR 1.81, 95% Cl 1.28 to 2.58, p<0.001, *I²*=85% |
| HM  vs  CM,  Placebo | Su (2009) | 46 RCTs, n=4,155 | OR 5.30, 95% Cl 4.38 to 6.41, p<0.00001, *I*²=22.2% | IBS subtype  IBS-D: 26 RCTs, n=2,347; OR 5.61, 95% Cl 4.33 to 7.25, p<0.00001, *I*²=30.1%  IBS regardless of sub-types: 20 RCTs, n=1,763; OR 4.95, 95% Cl 4.38 to 6.55, p<0.00001, *I*²=13.8% |
| HM  vs  Probiotics | Bu (2020) | 41 RCTs, n=3,207 | RR 1.24, 95% Cl 1.18 to 1.30, p<0.00001, *I*²=55% | 1. Version of Rome criteria   Rome II: 13 RCTs, n=1,066; RR 1.32, 95% CI 1.22 to 1.43, p<0.00001, *I*²=28%  Rome III: 27 RCTs, n=2,041; RR 1.20, 95% CI 1.15 to 1.26, p<0.00001, *I*²=28%   1. Treatment duration   < 4weeks: 7 RCTs, n=458; RR 1.11, 95% CI 1.00 to 1.24, p=0.04, *I*²=56%  4weeks-6months: 34 RCTs, n=2,669; RR 1.26, 95% CI 1.20 to 1.33, p<0.00001, *I*²=44%   1. Types of probiotics   single-strain probiotics: 12 RCTs, n=874; RR 1.14, 95% CI 1.04 to 1.25, p=0.005, *I*²=18%  multi-strain probiotics: n=2,333; RR 1.27, 95% CI 1.21 to 1.32, p<0.00001, *I*²=18% |
| Abdominal pain score | | | | |
| HM  (or HM+CM)  vs  CM | Li (2017) | 2 RCTs, n=160 | OR 5.69, 95% Cl 2.35 to 13.78, p=0.0001, *I*²=0% | NR |
|  | Dai (2018) | 14 RCTs, n=1,095 | SMD -1.27, 95% Cl -1.99 to -0.56, p=0.0005, *I*²=96% | NR |
|  | Zhou (2019) | 11 RCTs, n=809 | MD -0.41, 95% Cl -0.56 to -0.27, p<0.00001, *I*²=92% | Treatment duration  4 weeks: 8 RCTs, n=580; MD -0.43, 95% Cl -0.54 to -0.32, p<0.00001, *I*²=81%  8 weeks: 3 RCTs, n=229; MD -0.56, 95% Cl -1.21 to 0.08, p=0.087, *I*²=94% |
|  | Wang (2020) | 4 RCTs, n=286 | MD -0.61, 95% Cl -0.70 to -0.52, p<0.00001, *I*²=63% | NR |
|  | Yao (2021) | 10 RCTs, n=679 | MD -0.65, 95% Cl -0.73 to -0.57, p<0.00001, *I²*=83% | NR |
| HM  vs  Placebo | Xiao (2015) | 2 RCTs, n=502 | RR 4.34, 95% Cl 2.64 to 7.41, p<0.00001, I²=0.0% | NR |
|  | Zhu (2016) | 3 RCTs, n=377 | RR 1.95, 95% Cl 1.61 to 2.35, p<0.00001, *I*²=0% | NR |
|  | Zheng  (2021) | 3 RCTs, n=916 | RR 1.85, 95% Cl 1.59 to 2.14, p<0.001, *I²*=0% | NR |
| Recurrence rate | | | | |
| HM  vs  CM | Li (2013) | 8 RCTs, n=556 | OR 0.19, 95% Cl 0.12 to 0.29, p<0.00001, *I*²=46% | NR |
|  | Li (2017) | 3 RCTs, n=271 | OR 0.15, 95% Cl 0.08 to 0.27, p<0.00001, *I*²=0% | NR |
|  | Zhou (2019) | 3 RCTs, n=310 | OR 0.70, 95% Cl 0.25 to 1.96, p=0.50, *I*²=65% | NR |
| HM  vs  Probiotics | Bu (2020) | 5 RCTs, n=382 | RR 0.27, 95% Cl 0.18 to 0.40, p<0.00001, *I*²=0% | NR |
| Diarrhea score | | | | |
| HM  (or HM+CM)  vs  CM | Shi (2008) | 2 RCTs, n=201 | RR 1.14, 95% Cl 1.04 to 1.24, p=0.003,  χ^2^=0.41 (p=0.52) | NR |
|  | Dai (2018) | 8 RCTs, n=574 | SMD -1.10, 95% Cl -1.95 to -0.25, p=0.01, *I*²=95% | NR |
|  | Wang (2020) | 4 RCTs, n=286 | MD -0.26, 95% Cl -1.05 to -0.20, p=0.004, *I*²=88% | NR |
|  | Yao (2021) | 5 RCTs, n=357 | MD -0.63, 95% Cl -1.06 to -0.20, p=0.004, *I²*=91% | NR |
| HM  vs  Placebo | Zhu (2016) | 4 RCTs, n=442 | RR 1.87, 95% Cl 1.60 to 2.20, p<0.00001, *I*²=0% | NR |
| Abdominal distention score | | | | |
| HM  (or HM+CM)  vs  CM | Dai (2018) | 8 RCTs, n=625 | SMD -0.37, 95% Cl -0.73 to -0.01, p= 0.04, *I*²=79% | NR |
|  | Wang (2020) | 3 RCTs, n=226 | MD -0.88, 95% Cl -1.54 to -0.21, p=0.010, *I*²=91% | NR |
|  | Yao (2021) | 5 RCTs, n=371 | MD -0.37, 95% Cl -0.52 to -0.22, p<0.00001, *I²*=92% | NR |
| Frequency of defecation score | | | | |
| HM  vs  CM | Li (2017) | 2 RCTs, n=167 | OR 4.38, 95% Cl 1.93 to 9.93, p=0.0004, *I*²=0% | NR |
|  | Dai (2018) | 7 RCTs, n=586 | SMD -1.42, 95% Cl -2.19 to -0.65,  p= 0.0003, *I*²=94% | NR |
|  | Zhou (2019) | 6 RCTs, n=512 | MD -0.47, 95% Cl -0.58 to -0.35, p<0.00001, *I*²=77% | NR |
|  | Yao (2021) | 4 RCTs, n=242 | MD -0.75, 95% Cl -1.34 to -0.16, p=0.17, *I²*=40% | NR |
| IBS-SSS | | | | |
| HM  vs  Placebo | Xiao (2015) | 4 RCTs, n=223 | SMD -0.67, 95% Cl -0.94 to -0.40, p<0.00001, *I*²=0.0% | NR |
|  | Zhu (2016) | 5 RCTs, n=332 | SMD -1.01, 95% Cl -1.72 to -0.30, p=0.005, *I*²=88% | NR |
| Fecal property score | | | | |
| HM  vs  CM | Zhou (2019) | 11 RCTs, n=809 | MD -0.38, 95% Cl -0.48 to -0.27, p<0.00001, *I*²=89% | Treatment duration  4 weeks: 8 RCTs, n=580; MD -0.30, 95% Cl -0.40 to -0.20, p<0.00001, *I*²=89%  8 weeks: 3 RCTs, n=229; MD -0.75, 95% Cl -1.12 to -0.38, p<0.0001, *I*²=77% |
|  | Yao (2021) | 5 RCTs, n=290 | MD -0.36, 95% Cl -0.51 to -0.22, p<0.00001, *I²*=74% | NR |
| Stool form | | | | |
| HM  vs  CM | Li (2017) | 2 RCTs, n=174 | OR 4.96, 95% Cl 2.11 to 11.65, p=0.0002, *I*²=0% | NR |
| Total symptom score | | | | |
| HM  vs  CM | Zhou (2019) | 8 RCTs, n=510 | MD -2.75, 95% Cl -3.66 to -1.84, p<0.00001, *I*²=87% | NR |
| Pain threshold | | | | |
| HM  vs  Placebo | Zhu (2016) | 2 RCTs, n=74 | MD 54.53, 95% Cl 38.76 to 70.30, p<0.00001, *I*²=39% | NR |
| Defecation threshold | | | | |
| HM  vs  Placebo | Zhu (2016) | 2 RCTs, n=74 | MD 17.59, 95% Cl -4.60 to 39.77, p=0.12, *I*²=59% | NR |
| IBS-QoL | | | | |
| HM  vs  Placebo | Zhu (2016) | 2 RCTs, n=84 | MD -4.58, 95% Cl -14.29 to 5.13, p=0.36, *I*²=0% | NR |
| Adverse events rate | | | | |
| HM  vs  CM | Li (2013) | 6 RCTs, n=578 | OR 0.24, 95% Cl 0.09 to 0.65, p=0.005, *I*²=0% | NR |
|  | Zhou (2019) | 10 RCTs, n=773 | OR 0.26, 95% Cl 0.08 to 0.86, p=0.03, *I*²=0% | NR |
|  | Zheng  (2021) | 2 RCTs, n=1,032 | RR 1.06, 95% Cl 0.78 to 1.42, p=0.79, *I²*=0% | NR |
| HM  vs  Placebo | Tan (2020) | 12 RCTs, n=1,574 | RR 1.40, 95% Cl 0.91 to 2.16, p=0.12, *I*²=19% | NR |
|  | Zheng  (2021) | 7 RCTs, n=1,51 | RR 1.51, 95% Cl 1.14 to 2.00, p=0.004, *I²*=0% | IBS Subtypes  IBS-D: 5 RCTs, n=1,527; RR 1.37, 95% Cl 1.01 to 1.86, p=0.039, I²=0% |

HM: Herbal medicine, CM: Conventional medicine, NR: Not reported, IBS-SSS: Irritable bowel syndrome symptom severity score, IBS-QoL: Irritable bowel syndrome quality of life, RCT: Randomized controlled trial, MD: Mean difference, SMD: Standardized mean difference, OR: Odds ratio, RR: Risk ratio, Cl: Confidence interval, SLIS: Soothing the liver and invigorating the spleen, WKIS: Warming the kidney and invigorating the spleen *cure rate1: the main symptoms and signs disappeared or mostly disappeared, and the cure rate index was > 95%; the cure rate index = [(the sum of IBS symptom scores before treatment − the sum of IBS symptom scores after treatment)/the sum of IBS symptom scores before treatment] × 100%. †cure rate2: abdominal pain, abdominal distension, and other symptoms disappeared, defecation frequency and fecal mass were normal, defecation occurred 1-2 times per day with firm stool and no mucus
