# Supplementary material for: An Overview of Systematic Reviews of Herbal Medicine for Irritable Bowel Syndrome
Source: Front Pharmacol. 2022 May 18;13:894122. doi: 10.3389/fphar.2022.894122 (PMC9158123; doi:10.3389/fphar.2022.894122)
Supplement: Supplementary file 4 [file Table4.DOCX]

Table S4. Chemical compositions of single components in Tong Xie Yao Fang.

| **Scientific name** | **Chemical compositions** |
| --- | --- |
| Rhizome of *Atractylodes macrocephala* Koidz (Zhu et al., 2021) | Atractylenolide III, atractylenolide IV, 3-acetyl-atractylon, β-eudesmol, biatractylenolide II |
| Root of *Saposhnikovia divaricata* (Turcz.) Schischk (Yokosuka et al., 2017) | 3’-O-angeloylhamaudol, ledebouriellol, divaricatol, sec-O-methylvisammioside, (9Z)-heptadeca-1,9-diene-4,6-diyn-3-ol, (9Z)-heptadeca-1,9-diene-4,6-diyne-3,8-diol, (9Z)-1-methoxy-9-heptadecene-4,6-diyn-3-ol, (8E)-heptadeca-1,8-diene-4,6-diyne-3,10-diol, (8E)-10-hydroperoxy-1,8-heptadecadiene-4,6-diyn-3-ol |
| Peel of *Citrus reticulatae* Blanco. (Boughendjioua and Boughendjioua, 2017) | α-Thuyene, α-Pinene, sabinene, myrcene, α-Terpinene, limonene, β -phellandrene, γ-Terpinene, p- Cymene, octanal, decanal, linalol, terpinene-4-ol, l-Caryophyllene, α- Terpinenol, N-N-butylpyrrole, l-Caryophyllene, dimethylAnthranilate, germacrene-d, thymol, δ-muurolen, β-Cubebene, copaen, 2-isopropyl-5metylphenol |
| Root of *Paeonia lactiflora* Pall. (Tan et al., 2020) | Desbenzoyl paeoniflorin, D-catechin, paeoniflorin, albiflorin, oxypaeoniflorin, benzoylpaeoniflorin, gallolpaeoniflorin, galloylalbiflorin, 1’-O-galloyl sucrose, 6’-O- galloyl sucrose, 6-O- galloyl sucrose, benzoyloxypaeniflorin, paeonol, methyl gallate, gallic acid, paeoniflorin sulfonate, tetragalloyl glucose, monoterpene, glycosides sulfite |

**References**

Boughendjioua, H., and Boughendjioua, Z. (2017). Chemical composition and biological activity of essential oil of mandarin (Citrus reticulata) cultivated in Algeria. *Int. J. Pharm. Sci. Rev. Res.* 44, 179–184.

Tan, Y. Q., Chen, H. W., Li, J., and Wu, Q. J. (2020). Efficacy, Chemical Constituents, and Pharmacological Actions of Radix Paeoniae Rubra and Radix Paeoniae Alba. *Front. Pharmacol.* 11, 1–11. doi:10.3389/fphar.2020.01054.

Yokosuka, A., Tatsuno, S., Komine, T., and Mimaki, Y. (2017). Chemical constituents of the roots and rhizomes of Saposhnikovia divaricata and their cytotoxic activity. *Nat. Prod. Commun.* 12, 255–258. doi:10.1177/1934578x1701200229.

Zhu, Q., Lin, M., Zhuo, W., and Li, Y. (2021). Chemical constituents from the wild atractylodes macrocephala koidz and acetylcholinesterase inhibitory activity evaluation as well as molecular docking study. *Molecules* 26. doi:10.3390/molecules26237299.
